# Deciphering molecular mechanism of silver by integrated omic approaches enables enhancing its antimicrobial efficacy in *E*. *coli*

**DOI:** 10.1371/journal.pbio.3000292

**Published:** 2019-06-10

**Authors:** Haibo Wang, Aixin Yan, Zhigang Liu, Xinming Yang, Zeling Xu, Yuchuan Wang, Runming Wang, Mohamad Koohi-Moghadam, Ligang Hu, Wei Xia, Huiru Tang, Yulan Wang, Hongyan Li, Hongzhe Sun

**Affiliations:** 1 Department of Chemistry, The University of Hong Kong, Hong Kong, P. R. China; 2 School of Biological Sciences, The University of Hong Kong, Hong Kong, P. R. China; 3 CAS Key Laboratory of Magnetic Resonance in Biological Systems, State Key Laboratory of Magnetic Resonance and Atomic and Molecular Physics, National Centre for Magnetic Resonance in Wuhan, Wuhan Institute of Physics and Mathematics, Chinese Academy of Sciences, Wuhan, P. R. China; 4 School of Chemistry, Sun Yat-sen University, Guangzhou, P. R. China; 5 State Key Laboratory of Environmental Chemistry and Ecotoxicology, Research Centre for Eco-Environmental Sciences, Chinese Academy of Sciences, Beijing, P. R. China; 6 State Key Laboratory of Genetic Engineering, Zhongshan Hospital and School of Life Sciences, Fudan University, Shanghai International Centre for Molecular Phenomics, Collaborative Innovation Centre for Genetics and Development, Shanghai, P. R. China; 7 Singapore Phenome Center, Lee Kong Chian School of Medicine, Nanyang Technological University, Singapore; Max-Planck-Institut fur Terrestrische Mikrobiologie, GERMANY

## Abstract

Despite the broad-spectrum antimicrobial activities of silver, its internal usage is restricted, owing to the toxicity. Strategies to enhance its efficacy are highly desirable but rely heavily on the understanding of its molecular mechanism of action. However, up to now, no direct silver-targeting proteins have been mined at a proteome-wide scale, which hinders systemic studies on the biological pathways interrupted by silver. Herein, we build up a unique system, namely liquid chromatography gel electrophoresis inductively coupled plasma mass spectrometry (LC-GE-ICP-MS), allowing 34 proteins directly bound by silver ions to be identified in *Escherichia coli*. By using integrated omic approaches, including metalloproteomics, metabolomics, bioinformatics, and systemic biology, we delineated the first dynamic antimicrobial actions of silver (Ag^+^) in *E. coli*, i.e., it primarily damages multiple enzymes in glycolysis and tricarboxylic acid (TCA) cycle, leading to the stalling of the oxidative branch of the TCA cycle and an adaptive metabolic divergence to the reductive glyoxylate pathway. It then further damages the adaptive glyoxylate pathway and suppresses the cellular oxidative stress responses, causing systemic damages and death of the bacterium. To harness these novel findings, we coadministrated metabolites involved in the Krebs cycles with Ag^+^ and found that they can significantly potentiate the efficacy of silver both in vitro and in an animal model. Our study reveals the comprehensive and dynamic mechanisms of Ag^+^ toxicity in *E*. *coli* cells and offers a novel and general approach for deciphering molecular mechanisms of metallodrugs in various pathogens and cells to facilitate the development of new therapeutics.

## Abstract 2

The antimicrobial properties of silver are poorly understood. Tracking of the silver(I) proteome in Escherichia coli, in combination with metabolomics, bioinformatics and bioassays, delineates the dynamic antimicrobial actions of silver at a molecular level.

## Introduction

Silver compounds have been historically used for the treatment of burns, ulcerations, and infected wounds [1, 2]. Silver (Ag) and silver nanoparticles (AgNPs) are nowadays widely used in healthcare, food industry, hard surface materials, and textiles [3]. In particular, silver nitrate (AgNO_3_) has been recently demonstrated to boost antibiotic efficacy against gram-negative bacteria and restore susceptibility of a resistant bacterial strain to antibiotics in animal models [4]. This has aroused great interests for reuse of metal-based antimicrobials to cope with current crisis of antimicrobial resistance [5–8], which has put public health at an enormous risk [9]. However, the toxicity of silver is the main concern for its internal usage. Identification of molecular targets of silver could allow deeper understanding of its molecular mechanism of action, which in-turn extends its therapeutic applications [10].

Enormous efforts have been made toward understanding the molecular mechanism of action of silver [4, 11–14]. Most studies, however, were hypothesis driven, as no direct binding targets of silver could be uncovered systematically. Such studies also failed to provide a global view on the mode of action of silver. Although proteins and enzymes have long been believed as potential targets of the antimicrobial activity of silver [10, 14, 15], proteome-wide identification of such targets have not been achieved until now, owing to technical challenges. Quantitative proteomics only allows profiling up- and down-regulated proteins induced by silver [16–18], which might not serve as direct targets of silver. Several approaches, e.g., laser ablation inductively coupled plasma mass spectrometry (LA-ICP-MS) [19], synchrotron X-ray fluorescence spectrometry (SXFS) [20], and liquid chromatography (LC) combined with ICP-MS [21, 22] have been applied to detect metal-associated proteins in gels/tissues or explore unknown metalloproteomes in microbes. However, these methods suffer from either limited sensitivity or accessibility of the facility, which restrains extensive exploration of direct silver-targeting proteins in bacteria.

To depict a complete cellular response of an organism exposed to drugs, integration of multiple-omics approaches is necessary. As a complementary technique to transcriptomics and proteomics, metabolomics has been widely used to explore the cellular response of an organism toward stress [23–25]. Metabolomics involves identification, quantification, and comparison of the metabolome, the total repertoire of small molecules in different biological systems, thereby allowing delineation of the overall dynamic metabolic alterations associated with drug exposure [25]. Metabolic response is a strategy, by which host mounts to attenuate the alterations from the internal or external environment. The responsive metabolites can be used to reprogram the metabolome, leading to a targeted metabolic strategy to adapt or suppress such alterations, namely, reprogramming metabolomics [25]. Metabolome reprogramming represents a novel and pivotal strategy to enhance the therapeutic effects of antibiotics in recent years [26–29].

## Results and discussions

### Development of LC-GE-ICP-MS

We have previously developed a continuous-flow gel electrophoresis hyphenated with ICP-MS (GE-ICP-MS), allowing simultaneous separation and detection of bismuth and its associated proteins [30, 31]. However, the approach is limited by rather poor separation resolution, owing to the fact that only 1D gel column was used. Herein, we advance the approach by combining LC with GE-ICP-MS (LC-GE-ICP-MS), thereby proteins could be finely separated by LC and GE according to the differences on isoelectric points (pIs) and molecular weights (MWs), respectively (S1 Fig). Using this approach, major Ag^+^-binding proteins in the model microorganism *E*. *coli* treated with AgNO_3_ were separated, identified, and validated. With a sensitivity for detection of Ag^+^ bound to proteins at the picomole level (S2A and S2B Fig), the LC-GE-ICP-MS enabled the MWs, pIs of proteins, as well as silver contents contained in proteins to be mapped out simultaneously with high resolution.

**Fig 1:**
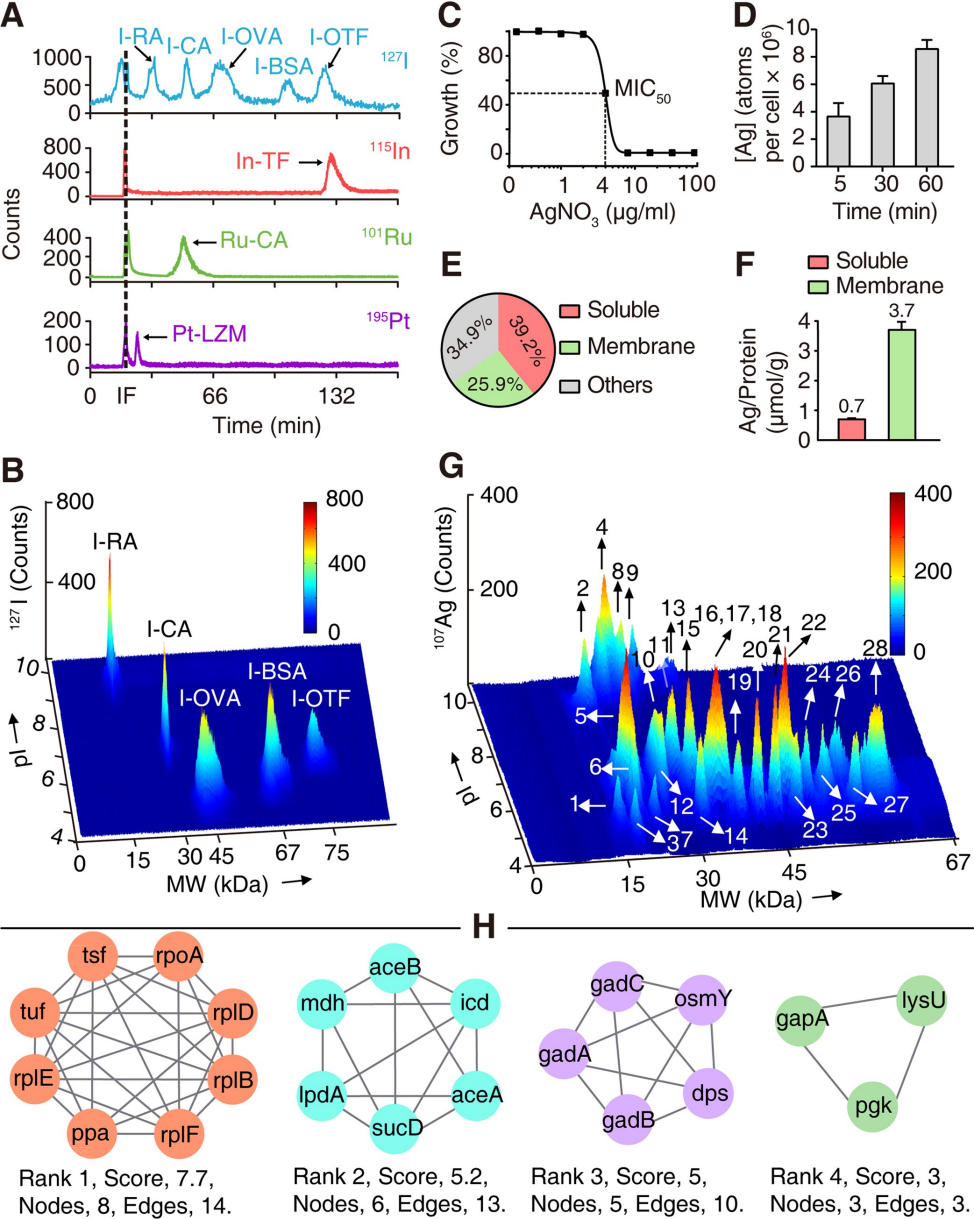
Illustration of the LC-GE-ICP-MS approach for exploration of Ag^+^-binding proteins in *E*. *coli*. (A, B) Validation of the feasibility of LC-GE-ICP-MS. (A) Labeling of proteins with metal ions and ^127^I. (B) Separation of ^127^I-labeled proteins by LC-GE-ICP-MS. (C) Antimicrobial activity of AgNO_3_ against *E*. *coli*. (D) Time-dependent uptake of silver in *E*. *coli* after treatment with 4 μg/ml AgNO_3_. (E) Distribution of silver in extracted fractions. (F) Silver to protein ratio in soluble and membrane proteins. For Fig 1E and 1F, *E*. *coli* cells were treated with 4 μg/ml AgNO_3_ for 1 h. (G) Map of Ag^+^-associated proteins in the *E*. *coli* cytosol. 1 (GrcA), 2 (ZraP), 3 (YgiW), 4 (RplF), 5 (FabA), 6 (OsmY), 7 (Ppa), 8 (RplE), 9 (RplD), 10 (GrxB), 11 (SodA), 12 (Dps), 13 (RplB), 14 (Tsf), 15 (SucD), 16 (Mdh), 17 (ProX), 18 (GapA), 19 (RpoA), 20 (Pgk), 21 (Tuf), 22 (Icd), 23 (AceA), 24 (LpdA), 25 (TnaA), 26 (GadA/B), 27 (LysU), 28 (AceB). (H) PPI of identified Ag^+^-binding proteins. Four functional categories are viewed by Cytoscape. Numerical values that underlie the graphs are shown in S1 Data. Ag, silver; AgNO_3_, silver nitrate; LC-GE-ICP-MS, liquid chromatography gel electrophoresis inductively coupled plasma mass spectrometry; MIC, minimum inhibitory concentration; PPI, protein-protein interaction.

To evaluate whether LC-GE-ICP-MS could achieve better resolution for protein separation than GE-ICP-MS, five proteins with distinct pIs and MWs were labeled with iodine (^127^I) [32] and examined by both systems. Three metal-bound proteins, i.e., platinum-bound lysozyme (Pt-LZM), ruthenium-bound carbonic anhydrase (Ru-CA), and indium-bound transferrin (In-TF), were used as internal standards to calibrate the MWs of ^127^I-labeled proteins (Fig 1A). Certain overlaps between proteins with similar MWs were observed in the analysis by GE-ICP-MS (Fig 1A), while the peaks corresponding to the ^127^I-labeled proteins were clearly and finely separated in the 3D graph of the LC-GE-ICP-MS analysis (Fig 1B), verifying the enhanced resolution of protein separation by this method.

#### Mapping silver proteome in *E*. *coli*

To explore the direct Ag^+^-binding proteins in *E*. *coli*, we first evaluated the bactericidal activity of AgNO_3_ against *E*. *coli* in the Luria-Bertani (LB) medium and found 4 μg/ml as a sublethal concentration (Fig 1C). Time-dependent uptake study showed that silver was up-taken rapidly in 5 mins, and the intracellular concentration of silver increased up to 1 h of exposure (Fig 1D), consistent with previous studies that Ag^+^ infiltrates the interior of *E*. *coli* shortly and interacts with cytoplasm primarily [33, 34]. We then measured the distribution of silver in different cellular components of *E*. *coli*. Accumulated silver was mainly distributed in proteins compared to that in nucleic acids (approximately 15%) [15, 35], with 39.2% ± 4.4% and 25.9% ± 3.6% in soluble and membrane proteins, respectively (Fig 1E), whereas around 5-fold higher Ag/protein ratio was found in the membrane protein fraction than in the soluble protein fraction (Fig 1F).

The signals of ^107^Ag peaks in 1D GE-ICP-MS increased with treatment time of silver up to 1 h when same amounts of extracted protein samples from both fractions (soluble and membrane proteins) were analyzed (S3A Fig). We then applied LC-GE-ICP-MS to explore Ag^+^-binding proteins in *E*. *coli* cytosol. As depicted in Fig 1G, we detected 28 ^107^Ag peaks corresponding to Ag^+^-associated proteins, mainly with MWs less than 50 kDa and pIs lower than 6.5. The protein eluates corresponding to each of the peaks were further fractionized, collected, and subjected to identification via peptide mass fingerprinting (S13 Table) after separation by 1D slab-gel electrophoresis. As the first dimensional separation is based on pI, proteins with similar or identical MWs but different pIs were well separated by LC-GE-ICP-MS (S3B Fig and S2 Table), such as GrcA (MW 14.3 kDa, pI 5.1) and ZraP (MW 15.2 kDa, pI 9.2); FabA (MW 19.1 kDa, pI 6.1) and RplF (MW 18.9 kDa, pI 9.7); Ppa (MW 19.8 kDa, pI 5.0) and RplE (MW 20.3 kDa, pI 9.5); RplD (MW 22.1 kDa, pI 9.8) and GrxB (MW 22.3 kDa, pI 6.3); RplB (MW 29.9 kDa, pI 10.9), Tsf (MW 30.5 kDa, pI 5.2), and SucD (MW 30.0 kDa, pI 6.1). Likewise, proteins with similar pIs but different MWs were well separated by the column GE system in the second dimension as well. Compared with the 1D GE-ICP-MS, by which only about 10 peaks were observed (S3A Fig), the LC-GE-ICP-MS achieved higher resolution for protein separation. Moreover, proteins with relatively low abundance could be enriched during the first dimensional separation by LC, allowing these proteins to be subsequently detected by GE-ICP-MS. Thus, our newly established LC-GE-ICP-MS exhibits the advantages of enhanced resolution and pre-enrichment capability. For the membrane proteins, five Ag^+^-binding proteins were resolved by 1D GE-ICP-MS (S3A Fig, S2 Table, and S14 Table). For *E*. *coli* without treatment of AgNO_3_, no silver peak was observed in the GE-ICP-MS profiles for both soluble and membrane proteins (S3C and S3D Fig).

Among the 34 identified proteins, only GrxB (PDB, 2MZC) and TnaA have been implicated to be bound by silver ions or nano-silver [36], the rest proteins are newly identified binding targets. To verify whether LC-GE-ICP-MS could precisely track Ag^+^-binding proteins, we extracted soluble proteins from gene knockout strains (Δ*zraP*, Δ*sucD*, Δ*mdh*, Δ*icd*, Δ*aceB*, and Δ*sodA*) after treatment with AgNO_3_ and examined whether the signals of Ag^+^-binding proteins in the corresponding MW window were still present. The results showed that the peaks corresponding to Ag^+^–Zrap, Ag^+^–SucD, Ag^+^–Mdh, Ag^+^–Icd, Ag^+^–AceB, and Ag^+^–SodA disappeared (S4A, S4B, S4C, S4D, S4E and S4F Fig). When we introduced plasmids containing the wild-type (WT) *zrap*, *sucD*, *mdh*, *icd*, *aceB*, and *sodA* into Δ*zraP*, Δ*sucD*, Δ*mdh*, Δ*icd*, Δ*aceB*, and Δ*sodA* mutants, respectively, the signals of Ag^+^-binding proteins in the corresponding MW window appeared again (S4A, S4B, S4C, S4D, S4E and S4F Fig). In contrast, no peak was observed at the corresponding MW window when the mutant plasmids containing *zrap*^CS^, *mdh*^3CS^, and *icd*^6CS^ (cysteine to serine) were transformed into corresponding gene knockout strains of Δ*zraP*, Δ*mdh*, and Δ*icd* (S4A, S4B and S4C Fig), confirming that mutation of silver-binding residues abolished silver-binding capability of these proteins *in vivo*.

Furthermore, we overexpressed and purified three proteins (Zrap, Mdh, and GapA) (S3, S4 and S5 Table) and examined their silver-binding capability upon incubation with 4 molar equivalents (eq.) of Ag^+^ by GE-ICP-MS. ^107^Ag peaks were observed at the MW around 15, 33, and 36 kDa, corresponding to Ag^+^–Zrap, Ag^+^–Mdh, and Ag^+^–GapA, respectively (S4G, S4H and S4I Fig). However, no ^107^Ag peaks were observed at the MW around 15, 33, and 36 kDa in the GE-ICP-MS profiles upon incubation of purified mutants of Zrap^CS^, Mdh^3CS^, and GapA^3CS^ with 4 eq. of Ag^+^ (S4G, S4H and S4I Fig). These results collectively corroborated that the newly developed approach can uncover authentic Ag^+^-bound proteins.

The abundance (presented by number of mRNA molecules/cell) [37] ranges from 0.69 to 54.6 for the identified 34 silver-binding proteins, and their Cys and His residues range from 1 to 10 and 1 to 13, respectively, suggesting that our method is not biased to only abundant proteins or those proteins with high Cys or His contents. Nonetheless, the method is not without limitation. It is unable to track [4Fe-4S] cluster–containing dehydratases that have been previously implicated as the targets of silver poisoning [11, 38], as silver poisoning leads to destruction of the [4Fe-4S] cluster and generation of the apo-enzyme. However, this does not hamper our understanding of silver toxicity. Indeed, combining the two sets of silver targets allows a comprehensive elucidation of the dynamics of Ag^+^ poisoning in *E*. *coli* cells (*vide infra*).

We next explored the biological insight of the identified Ag^+^-binding targets by Gene Ontology (GO) analysis with the Database for Annotation, Visualization, and Integrated Discovery (DAVID) [39]. In total, eight biological processes, six metabolic pathways, and four cellular components were significantly enriched (S5A, S5B, S5C, S5D, S5E and S5F Fig). These Ag^+^-binding proteins are mainly involved in TCA cycle (or Krebs cycle), glyoxylate cycle, glycolysis, translation, response to oxidative stress, and regulation of intracellular pH. We further calculated silver distribution in proteins involved in different biological processes and found the highest silver content in TCA cycle enzymes (S5G and S5H Fig). Analysis of protein–protein interaction (PPI) by Search Tool for the Retrieval of Interacting Genes/Proteins (STRING) [40] showed that these Ag^+^-binding proteins are enriched in four functional categories (Fig 1H). The largest numbers of Ag^+^-binding proteins were found in the network of translation, followed by TCA cycle, acid resistance, and glycolysis, suggesting that Ag^+^ targets multiple pathways in *E*. *coli*. Our results are in line with a recent report that Ag-resistant and -sensitive genes were found in multiple cellular systems using a chemical genetic screen. The common pathways (i.e., translation, acid resistance, TCA cycle, and oxidant detoxification) and genes (i.e., *mdh*, *gadA*, *sodA*, and *ompA*) confer silver cytotoxicity to *E*. *coli* were found both in our study and this report [41].

#### Metabolomics study

**Fig 2:**
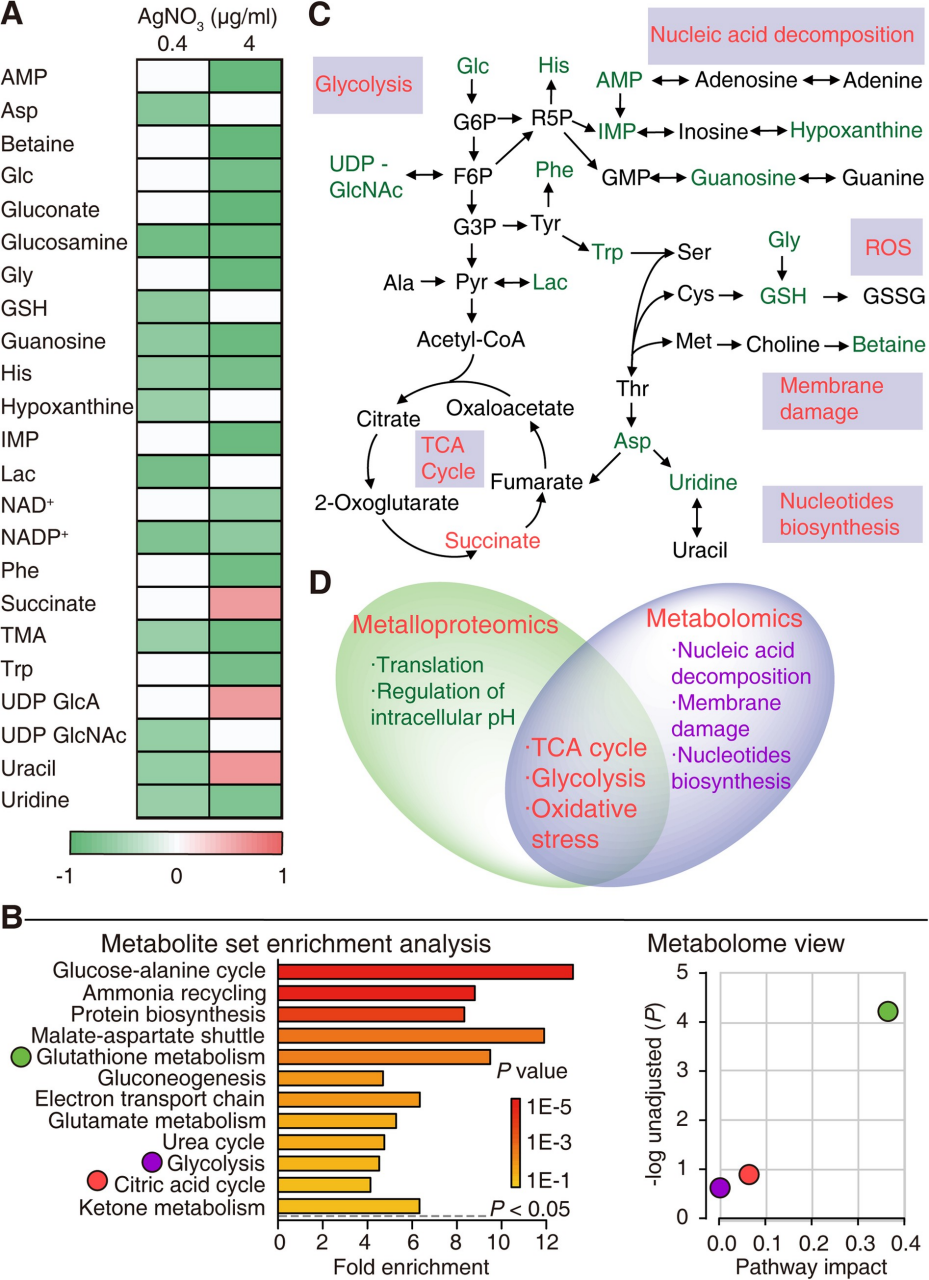
Depiction of metabolome changes in *E*. *coli* following Ag^+^ treatment. (A) Heat map of all changed metabolites in *E*. *coli* cells post to low (0.4 ***μ***g/ml) and high (4 ***μ***g/ml) concentrations of AgNO_3_. The metabolites colored in red and green represent significantly up or down alterations in the AgNO_3_-treated groups compared with control groups respectively (*n* = 10, *P* < 0.05). (B) Analysis of metabolic pathways disrupted after Ag^+^ treatment. Summary plots for the MSEA (left) are ranked by Holm *P* value. Metabolome view (right) shows key nodes in metabolic pathways that have been significantly altered upon Ag^+^ treatment. (C) Schematic summary of the metabolic response in *E*. *coli* exposed to Ag^+^. (D) Comparison of the functional pathways enriched by Ag^+^-binding proteins and altered metabolites induced by Ag^+^. Numerical values that underlie the graphs are shown in S1 Data. Ag, silver; AgNO_3_, silver nitrate; AMP, adenosine monophosphate; Asp, aspartate; Glc, glucose; Gly, glycine; GSH, reduced glutathione; His, histidine; IMP, inosine-5'-monophosphate; Lac, lactate; MSEA, Metabolite Set Enrichment Analysis; NAD^+^, nicotinamide adenine dinucleotide; NADP^+^, nicotinamide adenine dinucleotide phosphate; Phe, phenylalanine; TMA, trimethylamine; Trp, tryptophan; UDP GlcA, UDP glucoronate; UDP-GlcNAc, UDP-N-acetyl glucosamine.

To validate our findings, an NMR-based metabolomics study [23, 42] was carried out as a complementary approach to examine the systemic metabolic alterations of *E*. *coli* exposed to AgNO_3_ (S6 Table). Multivariate data analysis revealed changes in 23 metabolites out of 37 being unambiguously identified, and exposure of *E*. *coli* to a high dose of AgNO_3_ (4 μg/ml) resulted in more substantial metabolic changes (Fig 2A, S6 Table, and S7 Table). Consistent with the metalloproteomics analysis, levels of several metabolites in the glycolic pathway and TCA cycle were altered upon exposure to Ag^+^. In addition, the decline of glutathione (GSH) was observed, indicative of an elevated cellular oxidative stress induced by Ag^+^. The levels of adenosine monophosphate (AMP), inosine-5'-monophosphate (IMP), hypoxanthine, guanosine, and uridine, which are involved in nucleic acid degradation or synthesis, also decreased after Ag^+^ exposure (Fig 2C). Metabolite Set Enrichment Analysis (MSEA) [43] elaborated that over 10 enrichments were achieved (Fig 2B), with glutathione metabolism, glycolysis, and TCA cycle being the metabolic pathways significantly altered upon exposure to Ag^+^.

Our combined data from metalloproteomics and metabolomics suggest that Ag^+^ exerts its antimicrobial activity against *E*. *coli* by targeting the important metabolic and physiological pathways, including glycolysis, TCA cycle, and oxidative stress defense system (Fig 2D).

#### Silver primarily targets enzymes in glycolysis and the oxidative branch of TCA cycle

**Fig 3:**
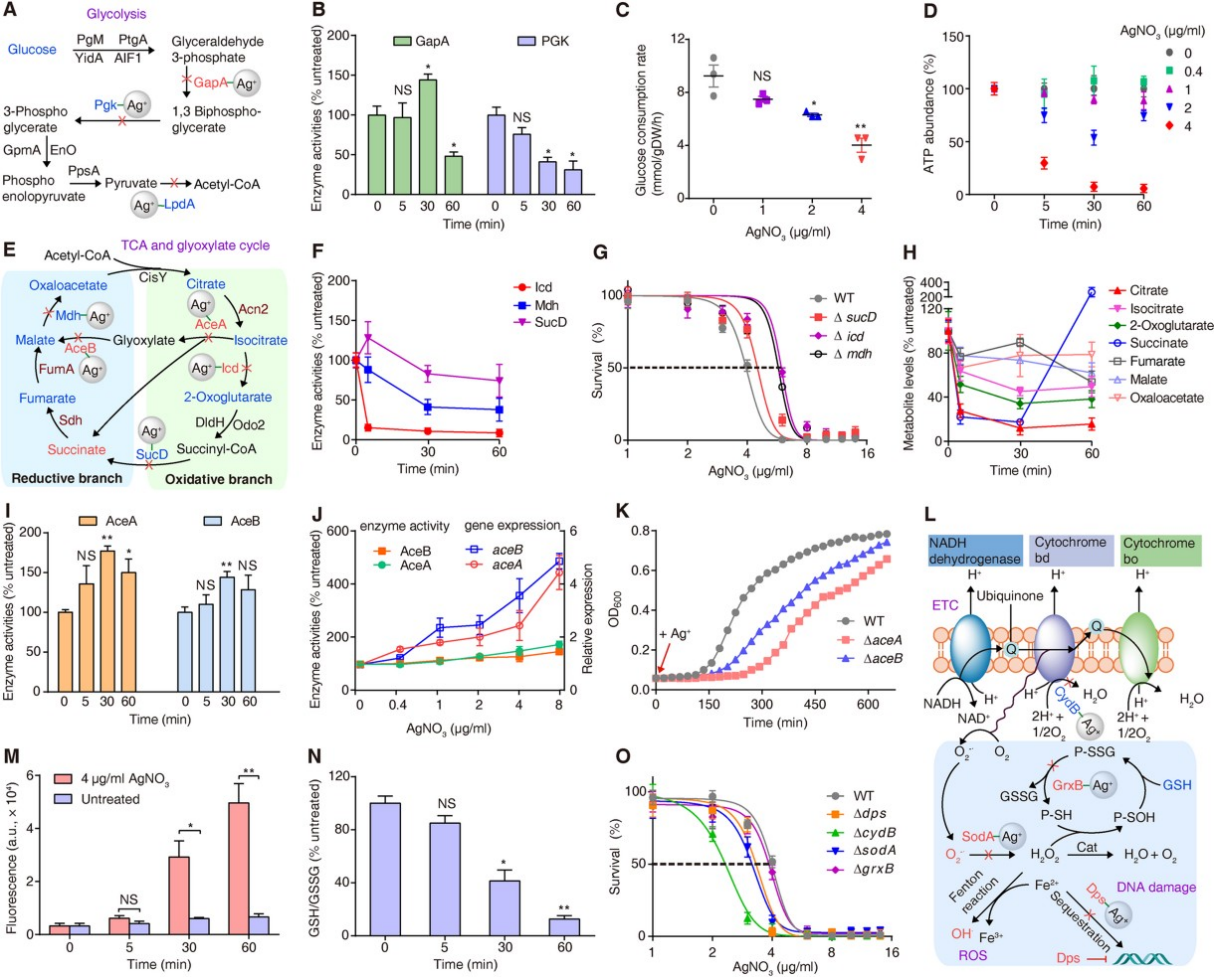
Silver targets glycolysis, TCA cycle, and oxidative stress defense system. (A) Diagram showing how silver disrupts glycolysis pathway. (B) Relative in vivo activities of GapA and Pgk (*n* = 3). (C) Glucose consumption rate of *E*. *coli* after treatment with AgNO_3_ at different concentrations (*n* = 3). (D) Relative ATP abundance (*n* = 3). (E) Diagram deciphering how silver disrupts TCA cycle and glyoxylate cycle. (F) Relative in vivo activities of Icd, Mdh, and SucD (*n* = 3). (G,O) Measurement of IC_50_ values of AgNO_3_ against WT *E*. *coli* and mutants (*n* = 3). (H) Relative concentration of metabolites (*n* = 3). (I) Relative in vivo activities of AceA and AceB (*n* = 3). (J) Relative expression and enzyme activities of AceA and AceB in *E*. *coli* after treatment with different doses of AgNO_3_. (K) Growth of WT *E*. *coli* and mutants Δ*aceA* and Δ*aceB* after treatment with 4 μg/ml AgNO_3_. (L) Diagram of the mechanism how silver affects oxidative stress defense system. (M) ROS levels (*n* = 3). (N) Relative GSH/GSSG ratio (*n* = 3). (B, F, H, I, M, N) All comparison were made between 4 μg/ml AgNO_3_ treated and control group at different time points. (A, E, L) The metabolites/intermediates and genes colored in red or blue are significantly up- or down-regulated after treatment of Ag^+^ for 1 h compared with untreated groups. *P < 0.05, **P < 0.01, and ***P < 0.001. NS (*P* > 0.05). Numerical values that underlie the graphs are shown in S1 Data. AceA, isocitrate lyase; AceB, malate synthase; AgNO_3_, silver nitrate; GapA, glyceraldehyde-3-phosphate dehydrogenase; GSH/GSSG, reduced glutathione/oxidized glutathione; Icd, isocitrate dehydrogenase; Mdh, malate dehydrogenase; NS, not significant; Pgk, phosphoglycerate kinase; ROS, reactive oxygen species; SucD, succinyl-CoA synthetase; TCA, tricarboxylic acid; WT, wild-type.

Three enzymes involved in glycolysis (Fig 3A), glyceraldehyde-3-phosphate dehydrogenase (GapA), phosphoglycerate kinase (Pgk), and dihydrolipoyl dehydrogenase (LpdA), were found to be bound by Ag^+^ in our metalloproteomics analysis (Fig 1G). We examined the in vivo activities of GapA and Pgk in *E*. *coli* after treatment with 4 μg/ml AgNO_3_ at different time points. We found that Ag^+^ inhibited over 50% activity of Pgk from 30 mins to 1 h, while inhibition on GapA to similar extent was observed at 1 h (Fig 3B). To further confirm that GapA and Pgk are real targets of Ag^+^ toxicity, we examined the activities of these two enzymes in the *E*. *coli* cell lysates with or without treatment of AgNO_3_. The result showed that addition of 100 μM AgNO_3_ to the lysate immediately abolished the activities of these two enzymes (S7 Fig). Since Ag^+^ binding inactivates these enzymes, the consumption of glucose by *E*. *coli* should be affected following the exposure to Ag^+^. To examine this, we supplemented glucose to the bacterial culture media and monitored its consumption by *E*. *coli* after exposure to different amounts of Ag^+^. As shown in Fig 3C, an Ag^+^-mediated dose-dependent inhibition on glucose consumption was observed relative to that in the untreated *E*. *coli* cells. Consistent with this, a significant and rapid dose-dependent depletion of adenosine triphosphate (ATP) was also observed upon treatment of the cells with AgNO_3_ (Fig 3D).

Several enzymes in the TCA cycle were found to be bound by Ag^+^ (Fig 3E), and the inhibition of these enzymes by silver was confirmed by the abolishment of their activities upon addition of AgNO_3_ to the extracted *E*. *coli* cell lysate (S7 Fig). To examine the primary targets of Ag^+^ amongst these targets, we further measured the in vivo activities of identified enzymes at different time points post treatment of 4 μg/ml AgNO_3_ (Fig 3F). The activity of isocitrate dehydrogenase (Icd) was inhibited by over 80% from 5 mins up to 1 h. A similar inhibition of malate dehydrogenase (Mdh) was observed but with slightly less extent (approximately 50%). The activity of succinyl-CoA synthetase (SucD) increased briefly upon Ag^+^ exposure but decreased afterward and displayed an overall trend of slight decrease. Collectively, these data indicate that the enzymes in the TCA cycle act as the primary targets of Ag^+^, with Icd and Mdh being inhibited most rapidly. We then constructed the corresponding gene knockout mutants in *E*. *coli*, i.e., Δ*icd*, Δ*sucD*, and Δ*mdh*, and examined their susceptibility to Ag^+^. Compared to the WT strain, variable and moderate growth defects were observed in these mutants in the early exponential phase (S8 Table). Higher minimum inhibitory concentration (MIC_50_) values in Δ*icd* and Δ*mdh* cells (6.0 and 5.7 μg/ml, respectively) than the WT *E*. *coli* (4.0 μg/ml) were observed (Fig 2G and S8 Table), demonstrating that Icd and Mdh are more prominent targets of silver ions. The observation of only moderate growth defect and increase of MIC_50_ in these individual deletion mutants is in well agreement with our metalloproteomics data that multiple targets are simultaneously inhibited by Ag^+^ during its poisoning of *E*. *coli* cells.

We further examined the dynamic alterations in the abundance of TCA cycle metabolites of *E*. *coli* cells following the exposure to Ag^+^ at different time points. As shown in Fig 3H, significant depletion of citrate, 2-oxoglugarate, and isocitrate was observed upon exposure to Ag^+^ for 1 h. Since these intermediates belong to the oxidative branch of the TCA cycle [44], these data suggest that this branch of the TCA cycle is primarily damaged by Ag^+^. On the other hand, inconsistent with the significant inactivation of Icd and Mdh activities shown above, we did not observe the accumulation of the corresponding substrates, i.e., isocitrate and malate (Fig 3H). This is not unexpected, since proteins containing Fe-S clusters were implicated as a class of Ag^+^ targets previously [11, 38], and the enzyme catalyzing isocitrate formation, aconitase (Acn2), and two consecutive enzymes upstream of malate formation, fumarase (FumA) and succinate dehydrogenase (Sdh), are [Fe-S] cluster proteins, and their damages can stall the production of isocitrate, fumarate, and malate. The pattern of succinate is distinct, as its level was reduced in the first 30 mins but was elevated afterward; such a pattern was also observed in the metabolomics analysis (Fig 2A).

To eliminate the indirect growth inhibition effect, we selected two antibiotics (ampicillin and kanamycin) and compared the metabolic changes of *E*. *coli* after treatment with Ag^+^ and antibiotics at the concentrations that inhibit *E*. *coli* growth by 50%. As our metalloproteomic study demonstrated that silver-bound proteins are enriched in glycolysis and TCA cycle, we therefore selected nine metabolites involved in these two pathways for comparison. As shown in S7 Fig, significant depletion of all the metabolites tested (except succinate) was observed upon exposure *E*. *coli* to Ag^+^ for 1 h. In contrast, a general increase in the examined metabolites was noted after treatment of the bacterium with ampicillin and kanamycin, in agreement with a previous report [45]. Such a discrepancy suggest that metabolic changes upon treatment of Ag^+^ are not due to indirect growth-related effects; instead, they are silver specific. Different from conventional antibiotics, silver kills *E*. *coli* through targeting multiple proteins and disrupting multiple pathways accordingly.

#### Silver induces metabolic divergence from TCA cycle to glyoxylate cycle

Although we identified isocitrate lyase (AceA) and malate synthase (AceB) as silver-bound proteins, the in vivo activities of AceA and AceB were increased in the first 30 mins following exposure to 4 μg/ml AgNO_3_ (Fig 3I). Notably, the concomitant decrease of Icd activity (Fig 3F) and increase of AceA and AceB activities (Fig 3I) suggests a metabolic divergence in *E*. *coli* from the TCA cycle to the glyoxylate cycle in response to Ag^+^ toxicity, which is further verified by the significant up-regulation of *aceA* and *aceB* genes (Fig 3J) as well as the accumulation of succinate (Fig 3H). We further measured the expression levels of *aceA* and *aceB* as well as the enzyme activities of AceA and AceB in *E*. *coli* after treatment with a series of concentrations of AgNO_3_ for 1 h. An Ag^+^-mediated dose-dependent increase of the expression levels of *aceA* and *aceB* were observed (Fig 3J). However, the in vivo activities of AceA and AceB were not proportionally increased (Fig 3J), suggesting that although an adaptive metabolic divergence to the glyoxylate cycle occurred in response to initial Ag^+^ exposure, enzymes in this pathway are also damaged by Ag^+^ in the continuous Ag^+^ toxicity, resulting in a largely futile metabolic divergence. This is further supported by the activity tests in cell lysates in which addition of Ag^+^ abolished the activities of AceA and AceB (S7 Fig).

To test whether the metabolic switch from TCA cycle to glyoxylate shunt could contribute to the cell’s tolerance to silver, we examined the growth of WT, Δ*aceA*, and Δ*aceB* strains after treatment of 4 μg/ml AgNO_3_ (Fig 3K). As shown in S8 Fig, the WT, Δ*aceA*, and Δ*aceB* strains display nearly identical growth curves in the absence of Ag^+^. However, addition of Ag^+^ resulted in clearly different growth curves for the three strains after addition of Ag^+^, with Δ*aceA* and Δ*aceB* cultures displaying a longer lag phase and a slower growth than the WT in the early log phase, demonstrating that the deletion of *aceA* and *aceB* affects the metabolic adaptation that contributes to the silver tolerance of *E*. *coli* in these growth phases. The fact that the mutant cultures reached almost the same optical density (OD_600_) values as the WT cells after 600 mins (Fig 3K) further supports the ultimate futility of the metabolic divergence in the late stage of continuous Ag^+^ toxicity. Collectively, these results demonstrate that the glyoxylate cycle provides an alternative pathway for metabolism of *E*. *coli* during Ag^+^ stress, which contributes to the silver tolerance of *E*. *coli* cells in the early stage but ultimately is futile to counteract Ag^+^ toxicity.

Taken together, we demonstrate a dynamic poisoning process in *E*. *coli* by Ag^+^, i.e., Ag^+^ first damages multiple TCA cycle enzymes, which leads to serious damage to the oxidative branch of the TCA cycle and then induces a metabolic divergence to the reductive glyoxylate pathway. The glyoxylate pathway contributes to the alleviation of Ag^+^ stress transiently but eventually is futile for *E*. *coli* cells exposed to continuous Ag^+^ toxicity. Among the enzymes involved in TCA cycle, three (Icd, SucD, and Mdh) are newly identified protein targets of Ag^+^ by LC-GE-ICP-MS, and silver-induced impairment of their functions are validated in this study. Together with the previously identified [4Fe-4S] cluster-containing targets Acn2, FumA, and Sdh, a rather complete and dynamic Ag^+^ poisoning of the central metabolic process of *E*. *coli* is unveiled for the first time.

#### Silver targets oxidative stress defense system in a later stage of its toxicity

Induction of oxidative stress has been widely deemed to contribute to bactericidal activity of silver [12, 17]. We observed an elevated level of succinate post exposure to Ag^+^ for 60 mins (Fig 3H and S6 Fig); this could lead to a hyperactivation of respiration and facilitate the electron flow in the electron transport chain (ETC) [46]. However, our metalloproteomics identified another target of Ag^+^ in this pathway: the terminal oxidase cytochrome BD oxidase subunit II (CydB) whose inactivation could trap electrons and induce reactive oxygen species (ROS) [46]. Indeed, we found that Δ*cydB* strain is more sensitive to Ag^+^ than the WT (Fig 3O) [4], confirming the deleterious effect of Ag^+^ on this protein. Furthermore, two proteins, i.e., SodA and Dps, which are involved in eliminating ROS or sequestration of Fe^2+^ and subsequently protecting DNA from oxidative damages [47], were identified to be bound by Ag^+^ (Fig 3L). We then measured the ROS levels in Ag^+^-treated and untreated *E*. *coli* at different time points using chloromethyl derivative of 2′, 7′-dichlorodihydrofluorescein diacetate (CM-H_2_DCFDA), a general oxidative stress indicator [48]. No obvious increase of ROS was observed in *E*. *coli* treated with Ag^+^ for 5 mins, but a burst of ROS was noted from 30 mins to 1 h (Fig 3M and S9A Fig). We further examined the GSH/GSSG ratio, for which the decreased ratio indicates cells being exposed to increased oxidative stress. A significant decrease in the GSH/GSSG ratio occurred after treatment with Ag^+^ for 30 mins, confirming that Ag^+^ induced ROS in a later stage of its toxicity (Fig 3N). It is conceivable that binding of Ag^+^ to antioxidant proteins SodA, Dps, and GrxB (Fig 3L) disrupts their functions, resulting in a further accumulation of ROS and consequently cell death. Consistent with this, Δ*sodA* and Δ*dps E*. *coli* cells were more sensitive to Ag^+^ than the WT (Fig 3O) [4].

Real-time quantitative polymerase chain reaction (qRT-PCR) [49] analysis revealed that the metalloproteomics findings and metabolomics changes were correlated with the gene expressions (S9B Fig). We found that most of the genes coding for enzymes in the glycolysis and TCA cycles, e.g., *pgk*, *lpdA*, *mdh*, and *sucD*, were down-regulated immediately upon treatment with Ag^+^, and the down-regulation remained up to 1 h, whereas the expression of glyoxylate cycle genes *aceA* and *aceB* were up-regulated, reflecting an adaptive strategy employed by *E*. *coli* to decrease the glycolysis and TCA cycles and divert the metabolism to glyoxylate cycle. In contrast, there were no significant changes in the expression levels of *sodA*, *dps*, and *grxB* at 5 mins upon exposure to Ag^+^, but a sharp up-regulation of *sodA*, *dps*, and *grxB* was observed afterward, reflecting a defense strategy of *E*. *coli* to the elevated ROS levels.

#### Metabolites potentiate the bactericidal efficacy of silver against *E*. *coli* in vitro and in an animal model

These newly discovered Ag^+^-binding proteins represent new targets that could be harnessed to enhance the bactericidal efficacy of Ag^+^. Given that Ag^+^ inhibits key enzymes in glycolysis and TCA cycles and down-regulates cellular respiration, thus promoting the respiration by supplementation of exogenous metabolites, should enhance the antimicrobial activity of Ag^+^ to *E*. *coli*. Since Ag^+^ disrupts the carbon and energy catabolism at different points along the TCA cycle and glycolysis, the potentiation effects should correlate with the level of inactivation of those enzymes.

**Fig 4:**
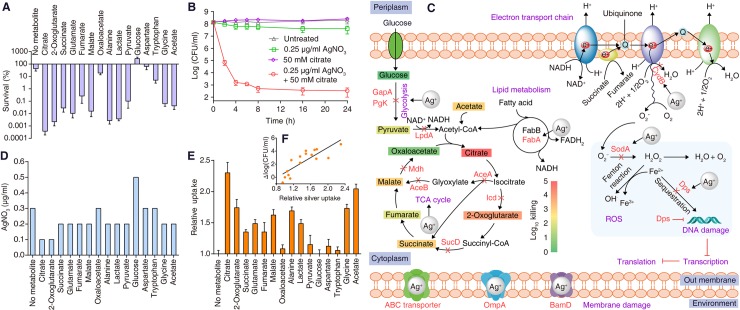
Metabolites enhance the bactericidal effect of Ag^+^ to *E*. *coli*. (A) Percentage survival of *E*. *coli* after addition of Ag^+^ and different metabolites (*n* = 3). (B) Time-dependent effects of cotreatment with Ag^+^ and metabolites on the viability of *E*. *coli*. Citrate was used as an example (*n* = 3). (C) The first snapshot showing how Ag^+^ kill *E*. *coli* by targeting multiple essential pathways at the molecular level. Metabolites are colored according to the killing they induced (Log_10_ killing). (D) MIC_90_ of AgNO_3_ without or with supplementation of metabolites. (E) Metabolite-enhanced uptake of silver by *E*. *coli* (*n* = 3). (F) The correlation of metabolite induced uptake of silver, and metabolite enabled enhancement of bactericidal efficacy of silver. Y = 3.86*X −3.16, R^2^ = 0.74. Numerical values that underlie the graphs are shown in S1 Data. Ag, silver; AgNO_3_, silver nitrate; MIC, minimum inhibitory concentration.

To test our hypothesis, we screened 15 metabolites involved in the TCA cycle, glycolysis, as well as other metabolites whose levels are decreased in our metabolomics study following Ag^+^ treatment for their ability to enhance the antimicrobial activity of Ag^+^ against *E*. *coli*. We examined the survival rates of *E*. *coli* after treatment of Ag^+^ together with each of the metabolites for 8 h in M9 minimal medium [28, 29]. As shown in Fig 4A, compared with the control (only Ag^+^-treated *E*. *coli* without addition of metabolites), the cell viability was significantly reduced by around three orders of magnitude when Ag^+^ was coadministered with selective metabolites involved in the TCA cycle, i.e., acetate, citrate, 2-oxoglutarate, succinate, fumarate, malate, and glutamate. Generally, combination of Ag^+^ and metabolites in the oxidative branch of TCA cycle showed more rapid and significant killing to *E*. *coli* (Fig 4A), with citrate exhibiting the highest potentiation effect, followed by 2-oxoglutarate, glutamate, and succinate, consistent with our findings that Ag^+^ exhibits the strongest inhibition or alteration to the enzymes or intermediates in the oxidative branch of the TCA cycle (Fig 4C). Meanwhile, the metabolites alone had negligible inhibition on the growth of *E*. *coli* (S10 Fig). In contrast, the metabolites involved in late steps of the TCA cycle exhibited less potentiation effect, with oxaloacetate showing no significant enhancement. However, malate exhibited over 1,000-fold potentiation, which could be explained by Ag^+^ binding and inhibition to Mdh and FumA in the TCA cycle. Besides, around two orders of magnitude reduction on the viability of *E*. *coli* were observed when metabolites that enter glycolytic pathway (alanine, pyruvate, and lactate) were supplemented. The fact that pyruvate has similar potentiation effect as citrate supports that the metabolic disruption by silver could be attributed to the pyruvate cycle, which has been recently identified as the energy-generated cycle in *E*. *coli* [26]. In contrast, a higher viability of the cell treated with Ag^+^ and glucose together than the control was observed (Fig 4A). Such a protective effect mediated by glucose might be attributed to glucose-induced efflux of Ag^+^ [50, 51]. No significant potentiation effects were observed by other down-regulated metabolites, i.e., aspartate and tryptophan.

It has been reported previously that treatment of *E*. *coli* cells with different antibiotics (ampicillin, kanamycin, and norfloxacin) led to higher levels of TCA cycle intermediates, such as citrate [45]. However, in this study, significant depletion of citrate was observed in *E*. *coli* upon exposure to Ag^+^. Despite that reprograming metabolomics has been employed to combat antibiotic resistance, metabolites are selected based on the metabolic response of the bacteria under stress, as the exogenous metabolites are used to reprogram the metabolome to adapt or suppress the metabolic changes [25]. For instance, metabolites that enter the upper glycolysis and pyruvate are able to induce rapid killing of persisters by gentamicin [28] while alanine, glucose, and glutamate exert higher potentiation effects for kanamycin to kill antibiotic-resistant bacteria [26, 29]. In our study, metabolites from the oxidative branch of the TCA cycle (or pyruvate cycle) generally show higher potentiation effect, whereas glucose exerts a protective effect when coadministrated with silver. Such discrepancies between silver and antibiotics clearly demonstrate that both the metabolic responses and metabolite-dependent potentiation strategy in our study are silver specific.

We further examined the time- and dose-dependent potentiation effects of these metabolites on Ag^+^. We found no recovery of the growth of *E*. *coli* after 24 h of cotreatment with Ag^+^ and effective metabolites (Fig 4B and S11 Fig). The metabolites within certain dose ranges exhibited a dose-dependent increased efficacy of Ag^+^ toward eradication of *E*. *coli*; specifically, citrate at a concentration as low as 0.1 mM could still potentiate the killing of Ag^+^ against *E*. *coli* significantly (S12 Fig). Consistently, we found that metabolites such as citrate and 2-oxoglutarate could reduce the MIC_90_ of AgNO_3_ from 0.3 μg/ml to 0.1 μg/ml, while acetate, succinate, glutamate, fumarate, malate, pyruvate, alanine, lactate, and glycine were less effective in this regard (Fig 4D).

We then measured the uptake of silver by *E*. *coli* after coadministration of metabolites and Ag^+^ for 1 h via ICP-MS. The highest uptake was found in the cells cotreated with citrate, followed by 2-oxoglutarate and other metabolites with a metabolite dose-dependent increase of Ag^+^ uptake (S13 Fig). The Ag^+^ uptake is generally in positive correlation with the potentiation ability of the metabolites (Fig 4E and 4F), implying that the increased uptake of Ag^+^ is partially responsible for the enhanced bactericidal efficacy. However, how different metabolites exert distinct potentiation effects on Ag^+^ still warrants further investigation.

We further evaluated the potential cytotoxicity of Ag^+^ in combination with different metabolites on human epithelial (HeLa) and human hepatoma G2 (HepG2) cells by the standard serial dilution method. Our results showed that all the metabolites at the concentration up to 50 mM exhibited no obvious cytotoxicity to HeLa and HepG2 cells (S14 and S15 Fig). Moreover, no synergistic cytotoxicity of the combination of metabolite and Ag^+^ was observed (S14 and S15 Fig). These data collectively indicate that the combination of Ag^+^ and metabolites exhibits significantly selective toxicity to bacteria over mammalian cells.

**Fig 5:**
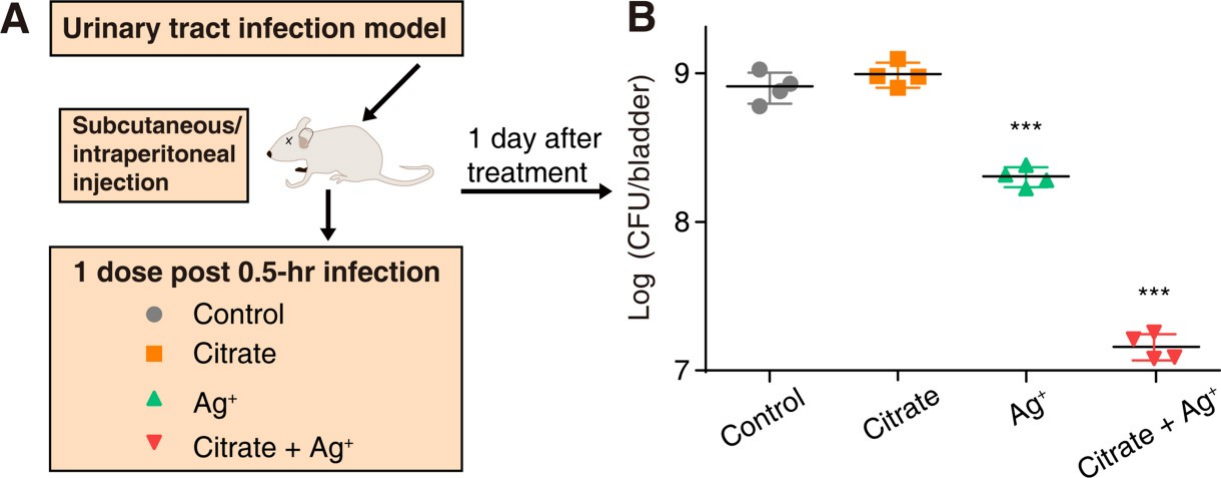
Citrate potentiates bactericidal activity of Ag^+^ against *E*. *coli* in a UTI mouse model. (A) Schematic illustration of the experimental protocol of the UTI mouse model. (B) CFU of *E*. *coli* counted in the bladder of infected mice 24 h after treatment with vehicle (*n* = 4), sodium citrate (1.5 g/kg), AgNO_3_ (0.75 mg/kg), and sodium citrate (1.5 g/kg) in combination with AgNO_3_ (0.75 mg/kg). ***P < 0.001. Numerical values that underlie the graphs are shown in S1 Data. Ag, silver; CFU, colony-forming unit; UTI, urinary tract infection.

We finally examined whether metabolites could potentiate bactericidal activity of Ag^+^ in a mouse urinary tract infection (UTI) model, one of the most commonly occurring clinical infections of *E*. *coli* [4]. Citrate was selected as a representative metabolite for the animal study, owing to its highest potentiation effect among the 15 metabolites in the in vitro study. The infection was performed by inoculating 2 × 10^8^ CFU of uropathogenic *E*. *coli* (UPEC) cells into the bladder of each mouse via transurethral catheterization (Fig 5). The infected mice received vehicle, monotherapy of AgNO_3_, sodium citrate, or combined therapy of AgNO_3_ and sodium citrate 0.5 h after infection. Animals were observed for another 24 h before sacrificed for colony counting. The treatment with sodium citrate alone resulted in slightly increased cell counts, whereas treatment with Ag^+^ reduced the cell counts by *ca*. 5-fold compared with the control group. Significantly, the combination of Ag^+^ and sodium citrate exhibited an enhanced bactericidal effect, with bacterial load reduced by *ca*. one order of magnitude over the Ag^+^-alone group (Fig 5). These results clearly demonstrate that metabolites can potentiate the antibacterial activity of Ag^+^ in vivo.

#### Conclusions

Silver has long been deemed to bind thiols in enzymes and thereby inactivate their functions [11, 52]; however, no direct binding protein targets have been identified and characterized systematically. Owing to the complexity of metal–protein interactions in cells, the lack of appropriate techniques to explore drug targets appears to be the main obstacle that hinders the understanding of molecular mechanism of silver as well as other metallodrugs. Using our homemade LC-GE-ICP-MS, we mapped out Ag^+^-binding proteins (Ag^+^-proteome) in *E*. *coli* for the first time. With the combined metalloproteomics, metabolomics, bioinformatics, and bioassays, we demonstrate that Ag^+^ disrupts multiple cellular processes in *E*. *coli*, particularly the TCA cycle, glycolysis, and oxidative stress defense systems. Further kinetic studies allow the first snapshot on how silver kills *E*. *coli* to be captured at the molecular level (Fig 4C). Ag^+^, once uptaken inside *E. coli* cell, primarily targets glycolysis and the oxidative branch of the TCA cycle (or pyruvate cycle) via functional disruption of the key enzymes, followed by adaptive glyoxylate cycle, and then abolishes bacterial oxidative defense capability through binding and inactivation of key antioxidant enzymes.

With broad-spectrum antimicrobial activity and less likelihood of resistance, silver has been thought to be an excellent candidate to tackle antimicrobial resistance [4]. However, its toxicity retards its wide application. Based on the molecular mechanism derived from our investigation, therapeutic effect of Ag^+^ could be enhanced by supplementation of metabolites from TCA cycle and glycolysis, thereby reducing its dosage. Our mouse model of *E*. *coli* infection successfully demonstrates a proof-of-principle that fundamental knowledge and molecular understanding of silver stress can be translated into new antibacterial approaches in clinic. Our study resolves long-standing questions on how Ag^+^ exerts its antimicrobial activity at the molecular level and may also open up new horizons for in-depth exploration of silver (and nanosilver) toxicology in other bacteria. Our initial data on Ag^+^-associated proteins in the gram-positive bacterium *Staphylococcus aureus* reveal that Ag^+^ has distinct targets in gram-negative and gram-positive bacteria. The integrated omic approaches we describe here can be widely applied to explore the molecular targets and mechanisms of action of other metal-based antimicrobial and anticancer drugs, thereby facilitating the development of new therapeutics.

## Materials and methods

### Ethics statement

All the animal experimental procedures were approved by the Committee on the Use of Live Animals in Teaching and Research (CULATR), the University of Hong Kong (approval no. 18–114). The experiments were conducted in accordance with Animals (Control of Experiments) Ordinance (Cap. 340), Department of Health, the Government of the Hong Kong Special Administrative Region.

### Materials and experimental design

*E*. *coli K12 MG 1655* is from our laboratory collection. Chromatography columns (Mono P 5/50 GL) and the FPLC system were from GE Healthcare. The column gel electrophoresis separation system was modified from a commercially available Mini Prep Cell system (Bio-Rad) by using the newly designed gel column (Φ = 2.5 mm) and replacing the solution-transferring tubing (PEEK tubing with Φ of 0.25 mm). Metal contents were determined by ICP-MS (Agilent 7700x). Peptide mass fingerprinting was performed on an LTQ Orbitrap VelosTM mass spectrometer (Thermo Fisher Scientific). All chemical reagents and standard proteins were purchased from Sigma-Aldrich, unless otherwise specified. All solutions were prepared from Milli-Q water (Milli-Q Ultrapure water systems, Millipore).

### Standard protein iodination

The iodination of standard proteins (ribonuclease A [RA], carbonic anhydrase [CA], ovalbumin [OVA], bovine serum albumin [BSA], and conalbumin [OTF]) were carried out according to a previous report [32]. In brief, for the iodination reaction, a 50 mM potassium iodide solution in Tris buffer (100 mM NaCl, pH 7.5) saturated with elemental iodine was used (termed as KI_3_). Protein solution was adjusted to a concentration of 1 mg/ml and incubated with KI_3_ at a final concentration of 5 mM for 10 mins at room temperature. The reaction was stopped by addition of sodium dithionite with a final concentration of 10 mM. The samples were washed with Tris buffer for three times to remove free iodide and then applied to SDS-PAGE and GE-ICP-MS to confirm the iodination. The molecular masses of iodinated proteins were recalibrated through the 1D slab gel electrophoresis to avoid the apparent mass shift resulted from any modification.

### Preparation of metal-bound proteins

Lysozyme (LZM) and CA at a concentration of 5 mM were incubated with cisplatin (*cis*-Pt(NH_3_)_2_Cl_2_) and RuCl_3_, respectively, at a molar ratio of 1:10 (protein:metal ions) at 4°C overnight [53]. Identical conditions were used to prepare Ag^+^-binding LZM. For preparation of indium (III)-bound human serum transferrin (TF), indium standard solution for ICP-MS supplemented with 50 mM NaHCO_3_ was used [54]. Unreacted In(III) were removed from the solutions with 3 kDa cut-off filters (Amicon) by centrifugation (Eppendorf) at 14,000 × g and 4°C for 20 mins, followed by two washing steps with Tris buffer. The successful labeling of metals to proteins (termed as Pt-LZM, Ru-CA, and In-TF) was further validated by GE-ICP-MS. The molecular masses of metal-bound protein makers were recalibrated through the 1D slab gel electrophoresis to avoid the apparent mass shift resulted from any modification.

### 2D method feasibility validation

#### First dimensional separation

Five ^127^I-labeled proteins were mixed and changed into starting buffer. Conditions utilized for LC were referred to standard procedures provided by the manufacture and further optimized. Polybuffers (GE Healthcare Life Sciences) were diluted 10-fold with milli-Q water before adjustment of pH. For pH in the range of 4–7, iminodiacetic acid was used to adjust pH. Bis-Tris (25 mM, pH 7.1) and polybuffer 74 (pH 4.0) were used as starting buffer and eluent, respectively. For pH in the range of 6–9, acidic acid was used to adjust pH of starting buffer (75 mM Tris) and eluent (polybuffer 96) to 10.0 and 6.0, respectively. The fractions of FPLC were collected and concentrated to the same volume for each 0.5 pH range and subsequently applied to GE-ICP-MS for further separation and detection.

#### Second dimensional separation

The apparatus and operation procedures were described in our previous report [30]. In this part, a reverse multilayer native resolving gel (13%→10%, with lengths of 1.2 and 1.8 cm, respectively) and 4% stacking gel of 0.5 cm were utilized to separate fractions from FPLC. A two-step voltage program with a stacking procedure of 60 min at 200 V and the resolving at 600 V was applied for the separation. The elemental detection by ICP-MS was started in the beginning of the second step. All conditions for GE-ICP-MS were kept the same for all samples. For each GE-ICP-MS experiment of ^127^I-labeled proteins, the same amounts of Pt-LZM, Ru-CA, and In-TF were added as internal standards for MW calibration. Ru-CA was selected for the calibration of intensity of iodine-labeled proteins.

### Media and growth conditions

*E*. *coli* cells from frozen stock were inoculated on Luria-Bertani (LB) agar plate and cultured at 37°C overnight. Single colonies were grown in LB broth for 16 h at 37°C. Cells were then diluted by 1:100 to LB media and grown for around 2 h to OD_600_ of 0.3. Different concentrations of AgNO_3_ were then added into culture. The viability of Ag^+^-treated and untreated cells was determined by counting CFUs followed by plating on LB agar plates and incubation overnight at 37°C. Cells after treatment of AgNO_3_ at different times were harvested and washed with cold phosphate-buffered saline (PBS) three times and subjected to further physiological tests.

### Silver uptake measurement

The culture of the bacteria was identical, as described in the growth conditions. Cells (OD_600_ of 0.3) were harvested after treatment with 4 μg/ml AgNO_3_ for 5, 30, and 60 mins. Bacterial pellets with same amounts of cell numbers (5 × 10^8^) were collected and digested with 100 μl 69.0% HNO_3_ (Fluka, 84385) overnight, which was diluted to a final volume of 1 ml with 1% HNO_3_. Samples were further diluted when the measured signals exceeded the liner range of standard curve. The standard curve of silver was prepared from multielement standard solution (90243, Sigma-Aldrich) for ICP-MS. Silver contents in bacteria were calculated according to the standard curve and normalized to number of atoms in single cells.

### Protein extraction and silver content measurement

AgNO_3_ of 4 μg/ml was used to treat *E*. *coli*. Ag^+^-treated cells were harvested at different time points by centrifugation (4,500 × g, 15 mins at 4°C) and washed with cold PBS three times. Collected pellets were resuspended in PBS buffer and then lysed through sonication (amplitude: 20%, 5 secs on, 20 secs off, in total 5 mins on the ice water bath). The mixed suspension after sonication was centrifuged to get the supernatant. The supernatant was further centrifuged (10 mins, 100,000 × g, and 4°C) and fractionated. Cytosolic proteins were collected from the supernatant, while the membrane proteins were dissolved from the pellets with PBS buffer containing 1% (w/v) SDS [55].

Protein concentrations and silver contents in cytosolic and membrane were detected by Pierce BCA Protein Assay Kit (Thermo Fisher Scientific) and ICP-MS, respectively. Silver content was normalized to protein concentration to yield the ratio of Ag/protein. For the distribution of silver in cytosolic and membrane fractions, same amounts of *E*. *coli* cells were prepared for measurement of total silver content; the percentages were obtained by dividing the silver content in different fractions to total silver content.

### Silver-binding protein separation and identification

For the first dimensional separation of *E*. *coli* proteins, conditions without specification were the same as the separation of ^127^I-labeled standard proteins but with several minor modifications to achieve higher resolution. In detail, 2% glycerol (v/v) was added to all starting buffers and eluents to increase the solubility of proteins. The polybuffers were diluted by 16-fold and the buffer with pH ranging from 3.5 to 6.5 was used, as the pIs of most proteins of *E*. *coli* were less than 6. The fractions of FPLC were collected and condensed to the same volume for each 0.5 pH range and subsequently applied to GE-ICP-MS for further separation and detection.

For the second dimensional separation with GE-ICP-MS, the concentration and lengths of freshly prepared column gel were optimized and all fractions from FPLC were subjected to the same separation conditions. Specifically, the lengths of 15% and 12% gels were increased to 1.7 and 2.0 cm, respectively, and the lengths of 4% stacking gel was increased to 1.2 cm. To avoid the interference of metals from the metal-bound proteins, ^127^I-labeled proteins were selected as internal standards for the calibration of MWs and the intensity of Ag^+^-binding proteins. For each GE-ICP-MS experiment, 0.5 μg I-RA, 1 μg I-CA, 2 μg I-OVA, 1 μg I-BSA, and 2 μg I-OTF were added.

To split the protein solutions after column gel electrophoresis, a T-connection connected with additional pump tubing with the same diameter was employed to transfer half of the eluted solutions to an automatic sample collection system. Eluents of 100 μl in each tube were collected. 1D slab gel electrophoresis was applied to separate the collected fractions after reducing the sample volume with an ultrafiltration device with a molecular weight cut-off of 3 kDa.

An additional silver determination procedure was applied to verify the associated silver in each band if multiple protein bands appeared in PAGE gel. The verified single band in the 1D SDS-PAGE gel was cut and digested for MS identification.

### Protein identification

The proteins in the collected fractions after gel electrophoresis separation were identified through peptide mass fingerprinting. In general, the proteins in gel pieces were extracted twice using 5% formic acid (FA)/50% acetonitrile (ACN) and then extracted once with 100% ACN. After purification by Ziptip (Millipore), the desalted peptides were mixed in a 1:1 ratio with 10 mg/ml α-cyano-4-hydroxycinnamic acid matrix (Fluka) dissolved in 0.1% FA/50% ACN.

The samples were injected via a nanospray chamber equipped with a gold-coated nanospray tip (New Objective). Protein identification and characterization were performed by using the 4800 MALDI TOF/TOF Analyser (ABSciex), which was equipped with a Nd:YAG laser that operates at 355 nm to ionize the samples. All mass spectra were acquired in positive-ion reflector mode using the 4000 series explorer version 3.5.28193 software (ABSciex). Each sample was analyzed with MALDI-TOF MS to create the peptide mass fingerprinting (PMF) data (scanning range of 900–4,000 m/z). The peak detection criteria for MS/MS used were an S/N of 5 and a local noise window width of 250 (m/z) and a minimum full-width half maximum (bins) of 2.9. The combined PMF and MS/MS search was then performed using GPS Explorer algorithm version 3.6 (ABSciex) against the nonredundant NCBInr database using the in-house MASCOT search engine version 2.2. The criteria for protein identification were based on the probability score of each search result. Significant matches had scores greater than the minimum threshold set by MASCOT (*P* < 0.05). All the results are presented in S13 Table.

### Bioinformatics analysis

DAVID (v 6.8, http://david.abcc.ncifcrf.gov/) was employed to analyze statistically over-represented GO terms among the identified Ag^+^-binding proteins [39]. The uploaded list is the gene names corresponding to the identified proteins. Fisher's exact test was used by DAVID to check significant over-representation (*P* < 0.05) of GO terms in the submitted data set against the *E*. *coli K12 MG 1655*. Benjamini multiple testing was performed to globally correct the *P* value controlling family-wide false discovery rate (*P* < 0.1). Data of over-represented biological process, Kyoto Encyclopedia of Genes and Genomes (KEGG) pathways, and cellular components are presented.

The STRING database (v9.1, http://string-db.org/) was applied to study the PPI of identified proteins [40]. A data set containing sequences of all the identified proteins was uploaded to STRING. The significantly enriched PPI networks were determined with the default parameter settings. The Cytoscape (v 3.4.0) was used for visualization of the interaction network among identified Ag^+^-binding proteins [56].

### Metabolomics study

Sample preparation and NMR analysis were performed as described previously [57].

#### Chemicals

D_2_O (99.9% in D) and sodium 3-trimethlysilyl (2,2,3,3-*d*_4_) propionate (TSP-d_4_) were obtained from Cambridge Isotope Laboratories (Miami, USA). Methanol, NaCl, NaH_2_PO_4_·2H_2_O, and K_2_HPO_4_·3H_2_O (all in analytical grade) were purchased from Sinopharm Chemical Reagent Co. Ltd. (Shanghai, China).

#### Bacterial culture

*E*. *coli* cells were cultured under the same conditions as in the protein identification part. AgNO_3_ at concentrations of 4 μg/ml and 0.4 μg/ml was added to the culture medium. Harvested cells were washed three times with cold PBS and then stored at −80°C until extraction. *E*. *coli* cells without treatment of AgNO_3_ were used as a control group.

#### Intracellular metabolites extraction

All of the frozen cell samples were homogenized in ice-cold water/methanol (1:2, v/v) extraction solution and subjected to freeze-thaw cycle three times. The cells were then sonicated in an ice water bath for 15 mins, with a sonication program of 1 min power on, following 1 min stop. The supernatants were collected via centrifugation for 10 mins at 12,000 × g and 4°C. The remaining pellets were further extracted twice with the same procedure. The supernatants were combined together and freeze-dried after removal of methanol in vacuum. The lyophilized cell extracts were weighed and dissolved into 600 μl phosphate buffer (0.1 M K_2_HPO_4_/NaH_2_PO_4_, pH 7.4) containing 90% D_2_O and 0.01% TSP-*d*_4_ for chemical shift calibration [58]. After vortexing and centrifugation (12,000 × g, 10 mins, 4°C), the supernatants of 550 μl were transferred into 5 mm NMR tubes for further analysis.

#### NMR spectroscopy

1D ^1^H NMR spectra of all the cell extracts were acquired at 25°C on a Bruker AVANCE III 600 MHz NMR spectrometer equiped with an inverse cryogenic probe (Bruker Biospin). A water-supressed standard 1D NMR spectrum was obtained for all samples via the first increment of NOESY pulse sequence, with a recycle delay of 2 s and mixing time of 80 ms. Typically, the 90° pulse length was about 10 μs, and 64 transients were recorded into 32,768 data points for each spectrum with a spectral width of 20 ppm.

To assign the NMR signals and identify metabolites for the samples, 2D NMR spectra, including ^1^H J-resolved, ^1^H−^1^H correlation spectroscopy (COSY), ^1^H−^1^H total correlation spectroscopy (TOCSY), ^1^H−^13^C heteronuclear multiple bond correlation spectra (HMBC), and ^1^H−^13^C heteronuclear single quantum correlation (HSQC), were acquired and processed, as described previously [59].

#### Data processing and multivariate data analysis

All free induction decays (FIDs) were multiplied by an exponential window function with a 1.0 Hz line broadening factor prior to Fourier transformation (FT). All spectra were phase and baseline corrected with reference to the peak of TSP at 0.00 ppm using Topspin (version 3.1, Bruker Biospin). The concentration of metabolites was calculated by deconvolution of the NMR spectra, compared to the internal standard TSP-*d*_4_, and then normalized to the wet weight of cells. Multivariate data analysis was performed using SIMCA-P^+^ (v 12.0, Umetrics). To obtain an overview of the data distribution, principal component analysis (PCA) was employed to examine group clustering and detect potential outliers. Orthogonal projection to latent structures discriminant analysis (OPLS-DA) was further constructed using the unit-variance scaling and validated by a 7-fold cross-validation method and CV-ANOVA approach (*P* < 0.05) [60, 61].

The loadings from the models were further transformed with color-scaled correlation coefficients of metabolites to show the difference and plotted using an in-house developed script in MATLAB 7.1 (The Mathworks, Inc.) [62]. In the loading plots, the color-coded correlation coefficients showed the variables contributed to the intergroup differentiation, with warmer colors representing metabolites with more significant contribution to the model than colder colored ones. A correlation coefficient of |r| > 0.602 was used in this study as the threshold value for the statistical significance based on the discrimination significance at the level of *P* < 0.05.

### Metabolite enrichment analysis

MSEA was performed with the web-based server (https://www.metaboanalyst.ca/) [63]. The summary plot for the MSEA is ranked by Holm *P* value with a cut-off value of 0.05. Holm *P* value is the *P* value adjusted by Holm–Bonferroni test that is a method to counteract the problem of multiple comparisons and is widely used for large-scale data analysis. The color code of the bar plot corresponds to the calculated *P* values. In the metabolic pathway impact analysis, the metabolome view shows key nodes in metabolic pathways that have been significantly altered. The y-axis represents unadjusted *P* value from pathway enrichment analysis. The x-axis represents increasing metabolic pathway impact according to the betweenness centrality from pathway topology analysis.

### Glucose consumption rate measurement

For the measurement of glucose consumption, 4 mg/ml glucose and different concentrations of AgNO_3_ were added into *E*. *coli* cell cultures (OD_600_ of 0.5). After treatment for 1 h, the bacteria were harvested, dried, and weighted. The concentrations of remained glucose in the supernatants were measured by using Glucose Assay Kit (Sigma-Aldrich) based on the glucose oxidase/peroxidase enzymatic reactions. A standard curve was simultaneously constructed using five concentrations of glucose in the range of 110 to 440 μM. The glucose consumption rates are normalized to the weight of the bacteria and expressed as mmol/gDW/h.

### ATP concentration detection

Cellular ATP concentrations of *E*. *coli* cells exposed to AgNO_3_ were measured with CellTiter-Glo Luminescent Cell Viability Assay (Promega) according to the manufacturer’s instruction. Generally, *E*. *coli* cells were harvested, washed with PBS, and diluted till 1 × 10^7^ cells/ml based on OD estimation. To a 100-μl aliquot of the culture, 100 μl of CellTiter-Glo reagent were added. The mixture was incubated at room temperature for 15 mins and then the luminescence was measured on PerkinElmer 2030. A standard curve was simultaneously constructed using six concentrations of ATP in the range of 0.125 to 1 μM.

### Enzymatic activity measurement

Colorimetric assay kits of glyceraldehyde 3-phosphate dehydrogenase (Abcam), succinyl-CoA synthetase (Abcam), malate dehydrogenase (Sigma-Aldrich), isocitrate dehydrogenase (Sigma-Aldrich) and isocitrate lyase (MyBioSource), malate synthase (Sigma-Aldrich), phosphoglycerate kinase (Creative enzymes) were used to measure corresponding enzymatic activities. For measurement of the in vivo enzymatic activities, *E*. *coli* cell cultures with or without treatment of AgNO_3_ were harvested, washed, resuspended, and lysed in lysis buffers with sonication. The protein concentration of supernatant after centrifugation was measured by BCA assay. Enzyme activities were measured according to the procedures provided by the manufactures. The enzyme activity was normalized to protein concentration.

For the measurement of the enzymatic activities in cell lysate, 100 μM of AgNO_3_ were added into cell lysate and incubated for 10 mins before the measurement.

### Metabolites content and GSH/GSSG ratio measurement

Contents of isocitrate, succinate, fumarate, and malate were detected by Isocitrate, Succinate, Fumarate, and Malate Colorimetric Detection Kits (Abcam), respectively. Citrate, 2-oxoglutarate, and oxaloacetate were detected through Citrate, 2-Oxoglutarate and Oxaloacetate Fluorometric Detection Kits (Abcam), respectively. GSH/GSSG ratio was measured according to the manufacture’s protocol with GSH/GSSG Ratio Detection Assay Kit (Fluorometric Green) (Abcam). In brief, *E*. *coli* cells were cultured to OD_600_ of 0.3 and then treated with 4 μg/ml AgNO_3_, 3 μg/ml ampicillin, and 6 μg/ml kanamycin. Cultures with or without treatment of AgNO_3_ or antibiotics were collected at the time points of 0, 5, 30, and 60 mins, washed, resuspended with cold PBS, and adjusted to OD_600_ of 3.0. Aliquots of 0.5 ml *E*. *coli* cells were sonicated and centrifuged. The resulting supernatant after deprotonation with 3 kDa cut-off filters (Amicon) was used for measurement of concentrations of metabolites and GSH/GSSG ratio.

### Gene knockout, growth curve measurement, and microdilution MIC assay

All *E*. *coli* gene deletion mutants were constructed by P1 phage transduction using the lysate of the donor strains from the KEIO knockout collection [64]. All the mutated strains were verified by colony PCR and DNA sequencing (BGI). The primers used for colony PCR are listed in S9 Table.

MIC values were determined by the standard broth microdilution method [4]. The MIC values of WT *E*. *coli MG1655* and mutants after treatment of AgNO_3_ were compared. In brief, WT *MG1655* and mutants were cultured in LB broth overnight at 37°C. The bacterial density was adjusted to around 1 × 10^6^ CFU/ml according to the measurement of optical density at 600 nm (OD_600_) and CFU counting on agar plates. AgNO_3_ was added into 96-well plates in triplicates and performed 2-fold serial dilution, followed by addition of bacterial inocula and further incubation at 37°C overnight. Wells without addition of AgNO_3_ were used as controls and wells with culture media only as background. The MIC_50_ was determined as the concentration that can inhibit 50% growth of the bacteria.

For the measurement of growth curve, single colony of WT *E*. *coli* or mutant strains were picked from LB agar plate and cultured in LB medium. After OD_600_ of the culture reached 0.5, the culture was diluted 10 times with fresh LB medium (OD_600_ = 0.05). The bacterial cultures were then incubated at 37°C in plate reader with orbital shaking at 220 rpm in a 24-well plate. The bacterial growth was monitored via OD_600_ values at 15-min intervals. For the Ag^+^-treatment, 4 μg/ml AgNO_3_ was added and the growth was monitored at 22-min intervals.

### Strains, plasmids, and primers for protein expression

Strains and plasmids used for protein overexpression are listed in S10 Table. The *E*. *coli* XL1-Blue and BL21 (DE3) strains harboring designed vectors were cultured in LB medium supplemented with appropriate concentration of ampicillin.

### DNA manipulation and plasmid construction

All the plasmids used as templates for PCR were extracted using the plasmid extraction kit (QIAprep Spin Miniprep kit, QIAGEN). All PCR primers were synthesized by BGI Company (Guangdong, China). Genes of *mdh*, *gapA*, *sucD*, *icd*, *sodA*, *aceB*, and *zrap* were amplified by PCR using *E*. *coli* MG1655 genomic DNA as a template with specific primer pairs containing AgeI and EcoRI restriction site at 5′- and 3′-end, respectively. The corresponding amplified products were digested with AgeI and EcoRI and ligated into pHisSUMO plasmid [65], which has been digested with the same restriction enzymes, to generate plasmids expressing Mdh, GapA, SucD, Icd, SodA, AceB, and Zrap, respectively. Site-specific mutagenesis was performed subsequently to generate Mdh, Icd, and Zrap variants carrying various point mutations (cysteine mutated to serine). The generated plasmids pHisSUMO-*mdh*, pHisSUMO-*mdh*^*3CS*^, pHisSUMO-*gapA*, pHisSUMO-*gapA*^*3CS*^, pHisSUMO-*zrap*, and pHisSUMO-*zrap*^*CS*^ were extracted and transformed into BL21 (DE3) cells for protein overexpression. The plasmids of pHisSUMO-*mdh* or pHisSUMO-*mdh*^*3CS*^; pHisSUMO-*sucD*, pHisSUMO-*icd*, or pHisSUMO-*icd*^*6CS*^; pHisSUMO-*sodA*, pHisSUMO-*aceB*, and pHisSUMO-*zrap*; or pHisSUMO- *zrap*^*CS*^ were transformed into MG1655 mutants Δ*mdh*, Δ*sucD*, Δ*icd*, Δ*sodA*, Δ*aceB*, and Δ*zrap*, respectively. All constructs were verified by DNA sequencing. Primers used in this study are listed in S11 Table.

### Protein expression and purification

Overnight cultures of BL21 (DE3) cells harboring pHisSUMO-*mdh*, pHisSUMO-*mdh*^*3CS*^, pHisSUMO-*gapA*, pHisSUMO-*gapA*^*3CS*^, pHisSUMO-*zrap*, and pHisSUMO-*zrap*^*CS*^ plasmids were diluted by 1:100 to fresh LB medium supplemented with 100 μg/ml ampicillin. Cells were grown at 37°C with rotation of 200 rpm until OD_600_ reached 0.6. MDH expression was induced by addition of isopropyl-β-D-thiogalactoside (IPTG) to a final concentration of 500 μM, and the bacteria were further incubated at 37°C for 3 h. For Zrap and GapA, protein expression was induced by addition of 200 μM of IPTG and further cultured for 16 h at 25°C. The bacteria were harvested by centrifugation (5, 000 g, 20 mins at 4°C), and the cell pellets were resuspended in Tris-HNO_3_ buffer (50 mM Tris-HNO_3_ buffer, 150 mM NaNO_3_, pH = 7.4) and lysed by sonication. The lysates were centrifuged at 15,000 g for 30 mins, the supernatant was collected and then applied to a 5 ml HisTrap Q column (GE Healthcare). The proteins were eluted with 300 mM imidazole in in Tris-HNO_3_ buffer. The eluted proteins were further subjected to SUMO protease (50 NIH units) cleavage at 25°C for 2 h to remove the His-tag and further purified by HiLoad 16/60 Superdex 200 column equilibrated with Tris-HNO_3_ buffer. The purified proteins were further confirmed by MALDI-TOF MS.

### RNA extraction and qRT-PCR

Quantitative qRT-PCR was performed with Superscript III (Invitrogen) according to the manufacturer’s instruction and by following the guidelines as described [66]. Bacterial total RNAs were extracted using RNeasy Mini Kit (Qiagen) as described by manufacturer’s protocol. To completely remove contaminated genomic DNA, the extracted RNA samples were subjected to DNase I treatment using the Turbo DNA Free Kit (Ambion). Absence of genomic DNA contamination was confirmed by PCR, with the prepared RNA as a template. The quantity and integrity of RNA were determined by using NanoDrop 2000 (Thermo Fisher Scientific) and verified by agarose gel electrophoresis.

Reverse transcription was performed using Super Script II reverse transcriptase (Invitrogen) and random hexamer primers (Invitrogen). RT-PCR reactions were carried out by using the SYBR Green qPCR Master Mix (Thermo Fisher Scientific) on StepOnePlus Real-Time PCR system (ABI). ΔΔC_T_ method was applied to quantify the transcription level of target genes by normalizing to the house-keeping gene *rrsA* (encoding ribosomal RNA 16S) and compared with the expression levels of the control group without treatment of AgNO_3_ [18). Each sample was assessed in three biological replicates and two technique replicates, including nontemplate control. The primers used for qRT-PCR are listed in S12 Table.

### GE-ICP-MS of purified proteins

Typical one dimensional GE-ICP-MS was employed to measure the silver-binding capability of the purified proteins. The concentration and length of freshly prepared column gel were optimized, and all the purified proteins were subjected to the same separation conditions. In brief, 2.5 cm 12% resolving gel and 0.6 cm 4% stacking gel were used. For each GE-ICP-MS test, 10 μl of 4 μM proteins were injected. ^127^I-labeled proteins were used as internal standards to calibrate MWs and intensity of Ag^+^-binding proteins.

### ROS detection

The ROS levels in *E*. *coli* was analyzed using chloromethyl derivative of CM-H_2_DCFDA (Thermo Fisher Scientific) [48]. *E*. *coli* cells (10^8^ cell/ml) after treatment with AgNO_3_ for different time were incubated with CM-H_2_DCFDA (final concentration of 10 μM) for 30 mins at 37°C in dark. Cells were then harvested by centrifugation and resuspended in PBS and transferred to 96-well plate. The levels of ROS were measured by florescence spectrophotometer (Beckman Coulter DTX880 multimode detector) with a setting of excitation wavelength (λ_ex_) of 491 nm and emission wavelength (λ_em_) of 518 nm. ROS levels in single *E*. *coli* cells were detected by flow cytometry with BD FACS AriaIII flow cytometer (Becton Dickinson).

### Metabolites and silver combined bactericidal assay

Metabolites potentiating the bactericidal effect of AgNO_3_ were performed as previously described [28, 29] with modifications. In brief, single *E*. *coli* colony was grown in LB broth for 16 hrs at 37°C with 200 rpm. Cells were then harvested and washed three times with PBS and resuspeneded in M9 minimal media, followed by cotreatment with metabolites and AgNO_3_. The potentiation effect of metabolites to AgNO_3_ in *E*. *coli* was evaluated by the survival rate of *E*. *coli*. 5-μl aliquots of cell cultures were removed, serially dilluted, and spot-plated onto LB agar plates to determine the survival rate. Percentage of survival was determined by dividing the CFU/ml of a sample at specific time point by the initial CFU/ml.

Silver uptake induced by metabolites was measured by ICP-MS. In brief, overnight-cultured *E*. *coli* cells were harvested and washed three times with PBS and resuspeneded in M9 minimal medium. AgNO_3_ and different metabolites were added to cultures and incubated for 1 h at 37°C, 200 rpm. Cells were then harvested and washed with PBS three times. The cell pellets were then digested with 100 μl 69.0% HNO_3_ overnight at 65°C, dilluted with 1% HNO_3_, and measured by ICP-MS.

### Cytotoxicity of silver in combination with metabolites to mammalian cells

HeLa cells and HepG2 cells were purchased from ATCC. The cells were grown in 96-well plates till 70%–80% confluency in DMEM and MEM medium, respectively. Combinations of different metabolites and AgNO_3_ were used to incubate the cells for 24 hrs. The cell viability was measured by Cell Proliferation Kit II (XTT) (Roche) according to the procedures provided by the manufacturer.

### UTI mouse model

All experiments were performed in accordance with the guidelines approved by Committee on the Use of Live Animals in Teaching and Research (CULATR), the University of Hong Kong. Six- to eight-week-old female BALB/c mice (approximately 18–22 g) were purchased from Charles River Laboratories, Inc. and used in all mouse studies. Animals were randomized to cages for each experiment.

The murine model of UTI was performed according to the method of a previous report [4], with modification. Static overnight cultures of type 1-piliated uropathogenic *E*. *coli* isolate were harvested and washed with PBS three times before further use. Mice were transurethrally inoculated with a dose of 2 × 10^8^ CFU of bacteria in aliquot of 50 μl PBS into their bladders using polyethylene tubing-covered 30gauge syringe*s*. Groups (*n* = 4 for each group) of mice received monotherapy of vehicle, AgNO_3_ (0.75 mg/kg), sodium citrate (1.5 g/kg), or combined therapy of AgNO_3_ and sodium citrate 0.5 h post infection. AgNO_3_ and sodium citrate were administrated intraperitoneally and subcutaneously, respectively. Mice administrated with vehicle were set as a control. All the mice were sacrificed 24 h post infection and bladders were collected in aliquot of 500 μl PBS and homogenized (Tissue Lyser II, QIAgen, Hilden, Germany) at a frequency of 30 Hz for 5 mins. The bacterial loads per bladder were numerated by agar plating. The homogenized bladder was serially diluted in PBS. 100 μl of each dilution were plated on LB agar plates and incubated at 37°C for 16 h. The colonies were counted, and the CFU/bladder was calculated.

### Statistical analysis

For all experiments without specified, three biological replicates and two technique replicates were performed. Two-tailed *t* test was used for all comparisons between two groups. Data are presented as mean ± SEM. *P < 0.05, **P < 0.01, and ***P < 0.001. Not significant (NS) (*P* > 0.05). Data of chemical shifts of identified metabolites and identification results of Ag^+^-binding proteins are included in the Supporting information.

## Supporting information

S1 Text Metabolite identification (related to Fig 2 in main text). (DOCX)

S2 Text Metabolic alterations induced by AgNO_3_ exposure (related to Fig 2 in main text). AgNO_3_, silver nitrate.
(DOCX)

S1 Fig Flow chart of experimental design (related to Fig 1 in main text). The design of LC-GE-ICP-MS is shown. Proteins were separated by LC according to the isoelectric points in the first dimension. Collected fractions from LC were subsequently subjected to the second dimensional separation by column GE. Silver signals were detected by ICP-MS. 1, cooling water; 2, eluent buffer; 3, protein solutions after separation by GE. GE, gel electrophoresis; ICP-MS, inductively coupled plasma mass spectrometry; LC, liquid chromatography.
(TIF)

S2 Fig Measurement of the sensitivity of GE-ICP-MS toward Ag^+^-binding proteins (related to Fig 1 in main text). (A) Standard curve of ^107^Ag measured with ICP-MS. (B) Measurement of the sensitivity of GE-ICP-MS to Ag^+^-binding proteins. A level of 6 picomole of Ag-LZM could be observed. Ag, silver; GE, gel electrophoresis; ICP-MS, inductively coupled plasma mass spectrometry; Ag-LZM, Ag-labeled lysozyme.
(TIF)

S3 Fig Separation of Ag^+^-binding proteins in *E. coli* (related to Fig 1 in main text). (A)1D GE-ICP-MS profiles of ^127^I-labeled standard proteins, Ag^+^-binding proteins in cytosol and membrane of *E*. *coli* at different time-points after treatment with 4 μg/ml AgNO_3_. (B) Separation of cytosolic proteins with LC (pH 7.4, left; pH 9.6, right). UV absorbance (280 nm), pH, and silver content are indicated. The silver contents and UV signals show a positive correlation. (C) Map of Ag^+^-associated proteins in the cytosol of *E*. *coli* without treatment with Ag^+^. (D) 1D GE-ICP-MS profile of Ag^+^-binding proteins in the membrane of *E*. *coli* without treatment with Ag^+^. No peaks corresponding to Ag^+^-binding proteins were observed in both cytosolic and membrane proteins. For all GE-ICP-MS experiments, one representative of three independent experiments is shown. Ag, silver; AgNO_3_, silver nitrate; GE, gel electrophoresis; ICP-MS, inductively coupled plasma mass spectrometry; LC, liquid chromatography.
(TIF)

S4 Fig Systemic validation of Ag^+^-binding proteins identified by LC-GE-ICP-MS (related to Fig 1 in main text). (A–F) Comparison of GE-ICP-MS profiles of Ag^+^-binding proteins in WT *E*. *coli* strain, its isogenic gene deletion mutants, and mutants containing the plasmids that express the corresponding genes (with or without site-specific mutagenesis). (A) Zrap. (B) Icd. (C) Mdh. (D) SucD. (E) SodA. (F) AceB. (G–I) GE-ICP-MS profiles of purified proteins with and without pre-incubation with Ag^+^. (G) Zrap. (H) Mdh. (I) GapA. Ag, silver; GE, gel electrophoresis; ICP-MS, inductively coupled plasma mass spectrometry; LC, liquid chromatography; WT, wild-type.
(TIF)

S5 Fig Distribution of silver among identified Ag^+^-binding proteins and bioinformatics analysis of these proteins (related to Fig 1 in main text). (A, B) Eight biological processes were significantly enriched by GO analysis (*P* < 0.05). Among them, TCA cycle is the one enriched with the highest significance (*P* = 5.2 × 10^−6^). Five identified Ag^+^-binding proteins are involved in TCA cycle. (C, D) Six signaling pathways were significantly over presented (*P* < 0.05). The TCA cycle is the one with the highest significance (*P* = 7.7 × 10^−5^). (E, F) Four cellular components were enriched. (G) Silver contained in identified proteins. (H) Distribution of silver in different pathways. (G, H) Representative results of three replicates. (I) PPIs. Ag, silver; GO, Gene Ontology; PPI, protein–protein interactions; TCA, tricarboxylic acid.
(TIF)

S6 Fig NMR-based metabolomic analysis (related to Fig 2 in main text). (A) Representative 600 MHz ^1^H NMR spectra of aqueous extracts from untreated, 0.4 μg/ml AgNO_3_-treated, and 4 μg/ml AgNO_3_-treated *E*. *coli* cells. Key: 1. Acetate; 2. Adenosine diphosphate (ADP); 3. Adenosine monophosphate (AMP); 4. Alanine (Ala); 5. Aspartate (Asp); 6. Betaine; 7. Formate; 8. Fumarate; 9. Glucose (Glc); 10. Glutamate (Glu); 11. Gluconate; 12. Glycine (Gly); 13. Guanosine; 14. Histidine (His); 15. Hypoxanthine; 16. Inosine; 17. Inosine-5'-monophosphate (5'-IMP); 18. Isoleucine (Ile); 19. Lactate; 20. Leucine (Leu); 21. Methanol; 22. Methionine (Met); 23. N-acetyl-glucosamine (GlcNAc); 24. Nicotinamide adenine dinucleotide (NAD^+^); 25. Nicotinamide adenine dinucleotide phosphate (NADP^+^); 26. Phenylalanine (Phe); 27. Reduced glutathione (GSH); 28. Succinate; 29. Trimethylamine (TMA); 30. Tryptophan (Trp); 31. Tyrosine (Tyr); 32. UDP glucuronate (UDP-GlcA); 33. UDP-N-acetyl glucosamine (UDP-GlcNAc); 34. Uracil; 35. Uridine; 36. Valine (Val); 37. Lysine (Lys). (B) O-PLS-DA scores plots (left) and coefficient-coded loading plots (right), discriminating between the untreated (green dots) and 0.4 μg/ml AgNO_3_ (low concentration) treated or 4 μg/ml AgNO_3_ (high concentration) treated *E*. *coli* cells (blue dots) (*n* = 10). These models are cross-validated with CV-ANOVA, *P* < 0.05. Metabolite keys corresponding to the numbers are shown in S6 Table. Detailed results of alterations in metabolites are presented in S7 Table.
(TIF)

S7 Fig Measurement of enzyme activities and metabolite relative abundance (related to Fig 3 in main text). A, the activities were measured in *E*. *coli* cell lysate with and without addition of 100 μM AgNO_3_ (*n* = 3). B, relative abundance of metabolites in *E*. *coli* after treatment with 4 μg/ml AgNO_3_, 3 μg/ml ampicillin, and 6 μg/ml kanamycin for 1 h (*n* = 3). Ctrl stands for untreated *E*. *coli* cells. Two-tailed *t* test was used for all comparisons between two groups. Data are presented as mean ± SEM. *P < 0.05, ** *P* < 0.01, and *** *P* < 0.001. NS (*P* > 0.05). Numerical values that underlie the graphs are shown in S1 Data. AgNO_3_, silver nitrate; NS, not significant.
(TIF)

S8 Fig Measurement of the growth of gene knockout strains and WT MG1655 without treatment of silver (related to Fig 3 in main text). (A) Δ*icd*, (B) Δ*mdh*, (C) Δ*sucD*, (D) Δ*aceA*, (E) Δ*aceB*, (F) Δ*grxB*, (G) Δ*sodA*, (H) Δ*dps*, (I) Δ*cydB*. Numerical values that underlie the graphs are shown in S1 Data. WT, wild-type.
(TIF)

S9 Fig Measurement of ROS levels with flow cytometry and relative gene expression levels with qRT-PCR (related to Fig 3 in main text). (A) CM-H_2_DCFDA fluorescence histogram of *E*. *coli* with (red) or without (blue) treatment of Ag^+^ (*n* = 3). Data are representatives of three replicates. (B) Relative gene expressions in *E*. *coli* treated with 4 μg/ml AgNO_3_ at different time points (*n* = 3). Gene expression was determined by qPCR and normalized against *rrsA* and untreated control. Two-tailed *t* test was used for all comparisons between two groups. Data are presented as mean ± SEM. *P < 0.05, ** *P* < 0.01, and *** *P* < 0.001. NS (*P* > 0.05). Numerical values that underlie the graphs are shown in S1 Data. Ag, silver; CM-H_2_DCFDA, chloromethyl derivative of 2′, 7′-dichlorodihydrofluorescein diacetate; NS, not significant; qRT-PCR, real-time quantitative polymerase chain reaction.
(TIF)

S10 Fig Growth of *E. coli* induced by metabolites (related to Fig 4 in main text). Gray lines represent untreated *E*. *coli*. (A) 50 mM citrate. (B) 50 mM 2-oxoglutarate. (C) 50 mM succinate. (D) 50 mM glutamate. (E) 50 mM fumarate. (F) 50 mM malate. (G) 50 mM oxaloacetate. (H) 50 mM alanine. (I) 50 mM lactate. (J) 50 mM pyruvate. (K) 50 mM glucose. (L) 10 mM aspartate. (M) 50 mM glycine. (N) 10 mM tryptophan. (O) 50 mM acetate. For each experiment, three biological replicates were performed. Data are presented as mean ± SEM. Numerical values that underlie the graphs are shown in S1 Data.
(TIF)

S11 Fig Effect of different metabolites on potentiation of bactericidal activity of Ag^+^ against *E. coli* at different time points (related to Fig 4 in main text). Gray, purple, green, and red lines represent control group; metabolite treated; 0.25 μg/ml AgNO_3_ treated; 0.25 μg/ml AgNO_3_ together with metabolite treated *E*. *coli* cells, respectively. (A) Survival of *E*. *coli* after treatment of different concentration of AgNO_3_ in M9 minimal medium. (B) 50 mM 2-oxoglutarate. (C) 50 mM succinate. (D) 50 mM glutamate. (E) 50 mM fumarate. (F) 50 mM malate. (G) 50 mM oxaloacetate. (H) 50 mM alanine. (I) 50 mM lactate. (J) 50 mM pyruvate. (K) 50 mM glucose. (L) 10 mM aspartate. (M) 50 mM glycine. (N) 10 mM tryptophan. (O) 50 mM acetate. For each experiment, three biological replicates were performed. Data are presented as mean ± SEM. Numerical values that underlie the graphs are shown in S1 Data. Ag, silver; AgNO_3_, silver nitrate.
(TIF)

S12 Fig Enhanced bactericidal efficacy of Ag^+^ by metabolites with different concentrations (related to Fig 4 in main text). *E*. *coli* cells were coadministered with 0.25 μg/ml AgNO_3_ and different metabolites for 8 hrs. (A) Citrate. (B) 2-Oxoglutarate. (C) Succinate. (D) Glutamate. (E) Fumarate. (F) Malate. (G) Oxaloacetate. (H) Alanine. (I) Lactate. (J) Pyruvate. (K) Glucose. (L) Aspartate. (M) Glycine. (N) Tryptophan. (O) Acetate. For each experiment, three biological replicates were performed. Data are presented as mean ± SEM. Numerical values that underlie the graphs are shown in S1 Data. Ag, silver; AgNO_3_, silver nitrate.
(TIF)

S13 Fig Dose-dependent study of metabolite-enabled silver uptake (related to Fig 4 in main text). *E*. *coli* cells were coadministered with 0.25 μg/ml AgNO_3_ and different metabolites for 1 h. (A) Citrate. (B) 2-Oxoglutarate. (C) Succinate. (D) Glutamate. (E) Fumarate. (F) Malate. (G) Oxaloacetate. (H) Alanine. (I) Lactate. (J) Pyruvate. (K) Glucose. (L) Aspartate. (M) Glycine. (N) Tryptophan. (O) Acetate. For each experiment, three biological replicates were performed. Data are presented as mean ± SEM. Numerical values that underlie the graphs are shown in S1 Data. Ag, silver; AgNO_3_, silver nitrate.
(TIF)

S14 Fig Cytotoxicity of Ag^+^ in combination with different metabolites to HeLa cells (related to Fig 4 in main text). HeLa cells were treated with serial concentrations of Ag^+^ and metabolites for 24 h. The cell toxicity was detected by Cell Proliferation Kit (XTT). All the metabolites showed no enhancement on the toxicity of Ag^+^ to human HeLa cells. (A) Citrate. (B) 2-Oxoglutarate. (C) Succinate. (D) Glutamate. (E) Fumarate. (F) Malate. (G) Oxaloacetate. (H) Alanine. (I) Lactate. (J) Pyruvate. (K) Glucose. (L) Aspartate. (M) Glycine. (N) Tryptophan. (O) Acetate. For each experiment, three biological replicates were performed. Data are presented as mean ± SEM. Numerical values that underlie the graphs are shown in S1 Data. Ag, silver; HeLa, human epithelial.
(TIF)

S15 Fig Cytotoxicity of Ag^+^ in combination with different metabolites to HepG2 cells (related to Fig 4 in main text). HepG2 cells were treated with serial concentrations of Ag^+^ and metabolites for 24 h. The cell toxicity was detected by Cell Proliferation Kit (XTT). All the metabolites showed no enhancement on the toxicity of Ag^+^ to human HeLa cells. (A) Citrate. (B) 2-Oxoglutarate. (C) Succinate. (D) Glutamate. (E) Fumarate. (F) Malate. (G) Oxaloacetate. (H) Alanine. (I) Lactate. (J) Pyruvate. (K) Glucose. (L) Aspartate. (M) Glycine. (N) Tryptophan. (O) Acetate. For each experiment, three biological replicates were performed. Data are presented as mean ± SEM. Numerical values that underlie the graphs are shown in S1 Data. Ag, silver; HeLa; human epithelial; HepG2, human hepatoma G2.
(TIF)

S1 Table Summary of standard proteins used in this study (related to Fig 1 in main text). (XLSX)

S2 Table Summary of identified Ag^+^-binding proteins from soluble fraction and membrane of *E. coli* (related to Fig 1 in main text). Ag, silver.
(XLSX)

S3 Table Peptide mass fingerprints of purified Zrap (related to Fig 1 in main text). Zrap, zinc resistance-associated protein.
(XLSX)

S4 Table Peptide mass fingerprints of purified Mdh (related to Fig 1 in main text). Mdh, malate dehydrogenase.
(XLSX)

S5 Table Peptide mass fingerprints of purified GapA (related to Fig 1 in main text). GapA, glyceraldehyde-3-phosphate dehydrogenase.
(XLSX)

S6 Table ^1^H and ^13^C chemical shifts of metabolites identified in *E. coli* (related to Fig 2 in main text). (XLSX)

S7 Table Significant changes in the levels of intracellular metabolites of *E. coli* cells upon exposure to AgNO_3_ at different concentrations (related to Fig 2 in main text). AgNO_3_, silver nitrate.
(XLSX)

S8 Table Determination of the MIC_50_ of AgNO_3_ against gene knockout strains and WT MG1655 (related to Fig 3 in main text). AgNO_3_, silver nitrate; MIC, minimum inhibitory concentration; WT, wild-type.
(XLSX)

S9 Table Colony PCR primer pairs in gene knockout study (related to Fig 3 in main text). PCR, polymerase chain reaction.
(XLSX)

S10 Table Strains, plasmids for protein expression (related to Fig 1 in main text). (XLSX)

S11 Table Primers for plasmid construction (related to Fig 1 in main text). (XLSX)

S12 Table qRT-PCR primer pairs (related to Fig 3 in main text). qRT-PCR, real-time quantitative polymerase chain reaction.
(XLSX)

S13 Table Summary of the peptide mass fingerprints of Ag^+^-binding proteins from soluble fraction of *E. coli* (related to Fig 1 in main text). Ag, silver.
(XLSX)

S14 Table Summary of the peptide mass fingerprints Ag^+^-binding proteins from E. coli membrane (related to Fig 1 in main text). Ag, silver.
(XLSX)

S1 Data All individual numerical values that underlie the summary data that are shown in Figs 1C, 1D, 1E, 1F, 2A, 3B, 3C, 3D, 3F, 3G, 3H, 3I, 3J, 3K, 3M, 3N, 3O, 4A, 4B, 4D, 4E, 4F, 5B, S7A, S7B, S8, S9, S10, S11, S12, S13, S14 and S15. (XLSX)

## Abbreviations

AceA: isocitrate lyase
AceB: malate synthase
Acn2: aconitase
Ag: silver
AgNO_3_: silver nitrate
AgNP: silver nanoparticle
AMP: adenosine monophosphate
CM-H_2_DCFDA: chloromethyl derivative of 2′, 7′-dichlorodihydrofluorescein diacetate
CydB: cytochrome BD ubiquinol oxidase subunit II
DAVID: Database for Annotation, Visualization, and Integrated Discovery
ETC: electron transport chain
FumA: fumarase
GapA: glyceraldehyde-3-phosphate dehydrogenase
GE: gel electrophoresis
GO: Gene Ontology
GSH/GSSG: reduced glutathione/oxidized glutathione
HeLa: human epithelial
HepG2: human hepatoma G2
^127^I: iodine
Icd: isocitrate dehydrogenase
ICP-MS: inductively coupled plasma mass spectrometry
IMP: inosine-5'-monophosphate
In-TF: indium-bound transferrin
LA: laser ablation
LB: Luria-Bertani
LC: liquid chromatography
LpdA: dihydrolipoyl dehydrogenase
Mdh: malate dehydrogenase
MIC: minimum inhibitory concentration
MSEA: Metabolite Set Enrichment Analysis
MW: molecular weight
OD: optical density
Pgk: phosphoglycerate kinase
pI: isoelectric point
PPI: protein–protein interaction
Pt-LZM: platinum-bound lysozyme
qRT-PCR: real-time quantitative polymerase chain reaction
ROS: reactive oxygen species
Ru-CA: ruthenium-bound carbonic anhydrase
Sdh: succinate dehydrogenase
STRING: Search Tool for the Retrieval of Interacting Genes/Proteins
SucD: succinyl-CoA synthetase
SXFS: synchrotron X-ray fluorescence spectrometry
TCA: tricarboxylic acid
UPEC: uropathogenic *E*. *coli*
UTI: urinary tract infection
WT: wild-type
