# Supplementary material for: Deciphering molecular mechanism of silver by integrated omic approaches enables enhancing its antimicrobial efficacy in E. coli
Source: PLoS Biol. 2019 Jun 10;17(6):e3000292. doi: 10.1371/journal.pbio.3000292 (PMC6557469; doi:10.1371/journal.pbio.3000292)
Supplement: S1 Text — (DOCX) [file pbio.3000292.s001.docx]

**S1 Text. Metabolite identification.**

Typical one-dimensional ^1^H NMR spectra from the extracts of *E. coli* cells with and without treatment of AgNO_3_ were obtained (S6A Fig). Metabolites were identified according to a previous report [1] and further confirmed by a range of 2D NMR spectra, including COSY, TOSCY, HMBC and HSQC. Assigned metabolites, together with their proton chemical shifts, multiplicities, and carbon chemical shifts are summarized in S6 Table. A total of 37 metabolites, including a range of amino acids, amino sugar, glucose, organic acids such as lactate and acetate, nucleosides and nucleotides, such as adenosine 5'-monophosphate (AMP), inosine-5'-monophosphate (IMP), hypoxanthine, guanosine, uridine, uracil, adenosine diphosphate (ADP), nicotinamide adenine dinucleotide (NAD^+^), NADP^+^, and membrane metabolites, such as betaine, trimethylamine (TMA) were identified. Those metabolites are mainly involved in glycolytic pathway and TCA relevant energy process, osmoregulation, oxidative stress as well as the metabolism of amino acid, nucleotides, and lipid. Multivariate data analyses including PCA and O-PLS-DA were further performed to obtain AgNO_3_-induced metabolic alterations (S7 Table).

1. Zhang L, Wang L, Hu Y, Liu Z, Tian Y, Wu X, et al. Selective metabolic effects of gold nanorods on normal and cancer cells and their application in anticancer drug screening. Biomaterials. 2013; 34(29), 7117-7126. doi: doi.org/10.1016/j.biomaterials.2013.05.043.
