# Supplementary material for: Deciphering molecular mechanism of silver by integrated omic approaches enables enhancing its antimicrobial efficacy in E. coli
Source: PLoS Biol. 2019 Jun 10;17(6):e3000292. doi: 10.1371/journal.pbio.3000292 (PMC6557469; doi:10.1371/journal.pbio.3000292)
Supplement: S2 Text — AgNO3, silver nitrate. (DOCX) [file pbio.3000292.s002.docx]

**S2 Text. Metabolic alterations induced by AgNO_3_ exposure.**

The OPLS-DA scores plots (S6B Fig) of the ^1^H NMR spectra manifested clear difference between the profiles of Ag^+^-treated and untreated *E. coli* cells. Ag^+^ induced metabolic alterations were further verified by constructing OPLS-DA models and the quality of the models was evaluated by the values of *R^2^* and *Q^2^*, which represent the quality of fit and predictability of the model respectively (S7 Table). The models were further cross-evaluated via a CV-ANOVA approach (*P* < 0.05) and permutation tests. The dominant metabolites in *E. coli* cells relevant to Ag^+^ treatment was annotated in the OPLS-DA coefficient plots (S6B Fig) and summarized in S7 Table. The absolute value of coefficient |r| indicates the significance of altered metabolites.

Compared to the untreated *E. coli* cells, treatment of cells with low concentration of AgNO_3_ induced a reduction in the levels of aspartate, histidine, lactate, glucosamine, UDP GlcNAc, NADP^+^, uracil, uridine, guanosine, hypoxanthine, trimethylamine and GSH. Both similarities and differences were observed for *E. coli* in response to low and high concentrations of AgNO_3_. Exposure to high concentration of AgNO_3_ induced more profound depletion in the levels of histidine, glucosamine, uridine, guanosine and trimethylamine, but the concentration of NADP^+^ decreased less. The levels of tryptophan, phenylalanine, glucose, AMP, gluconate, NAD^+^, IMP, betaine and glycine were depleted only for cells treated with high but not low concentration of AgNO_3_. Significant elevation in the levels of succinate, UDP GlcA and uracil were observed after exposure cells to high concentration of AgNO_3_. The disrupted metabolites are mainly involved in energy metabolism, TCA cycle, oxidative stress, nucleic acid metabolism (both decomposition and biosynthesis), cell membrane integrity and amino acid metabolism.
