# Supplementary figures and images for: Deciphering molecular mechanism of silver by integrated omic approaches enables enhancing its antimicrobial efficacy in E. coli
Source: PLoS Biol. 2019 Jun 10;17(6):e3000292. doi: 10.1371/journal.pbio.3000292 (PMC6557469; doi:10.1371/journal.pbio.3000292)

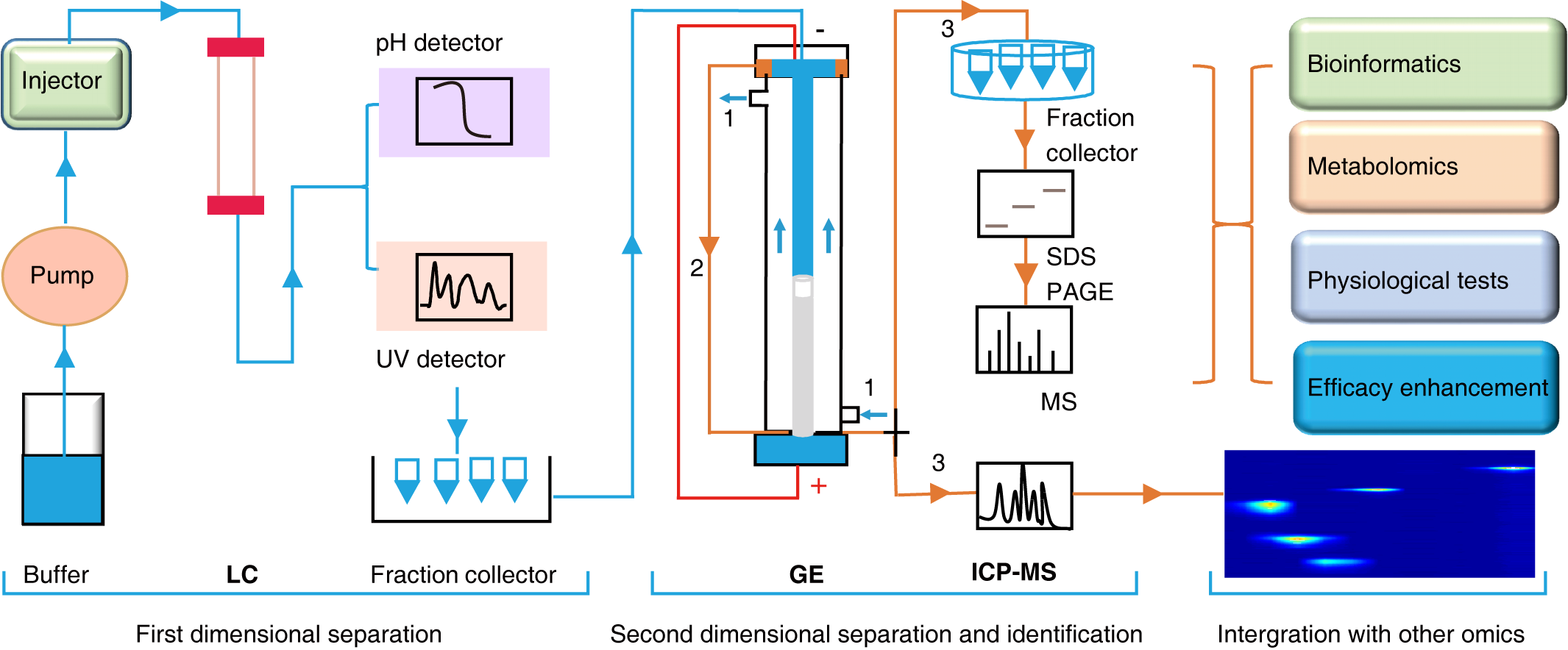

Supplement: S1 Fig — The design of LC-GE-ICP-MS is shown. Proteins were separated by LC according to the isoelectric points in the first dimension. Collected fractions from LC were subsequently subjected to the second dimensional separation by column GE. Silver signals were detected by ICP-MS. 1, cooling water; 2, eluent buffer; 3, protein solutions after separation by GE. GE, gel electrophoresis; ICP-MS, inductively coupled plasma mass spectrometry; LC, liquid chromatography. (TIF) [file pbio.3000292.s003.tif]

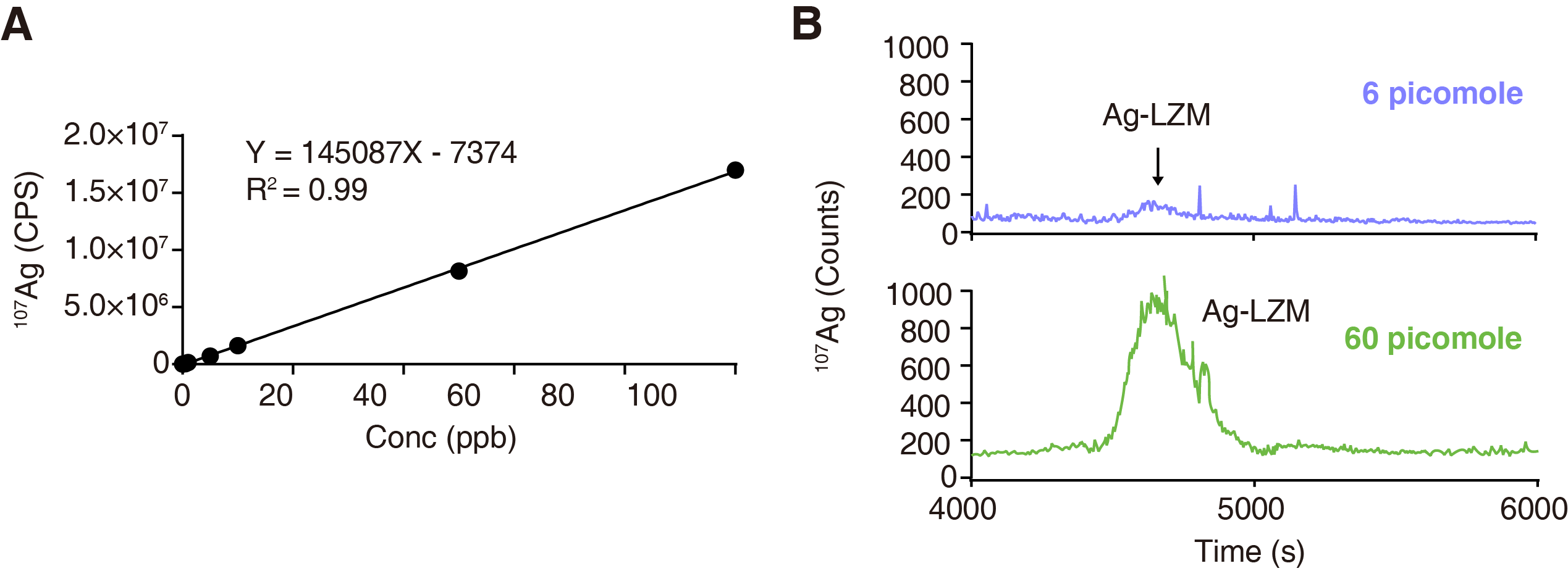

Supplement: S2 Fig — (A) Standard curve of 107Ag measured with ICP-MS. (B) Measurement of the sensitivity of GE-ICP-MS to Ag+-binding proteins. A level of 6 picomole of Ag-LZM could be observed. Ag, silver; GE, gel electrophoresis; ICP-MS, inductively coupled plasma mass spectrometry; Ag-LZM, Ag-labeled lysozyme. (TIF) [file pbio.3000292.s004.tif]

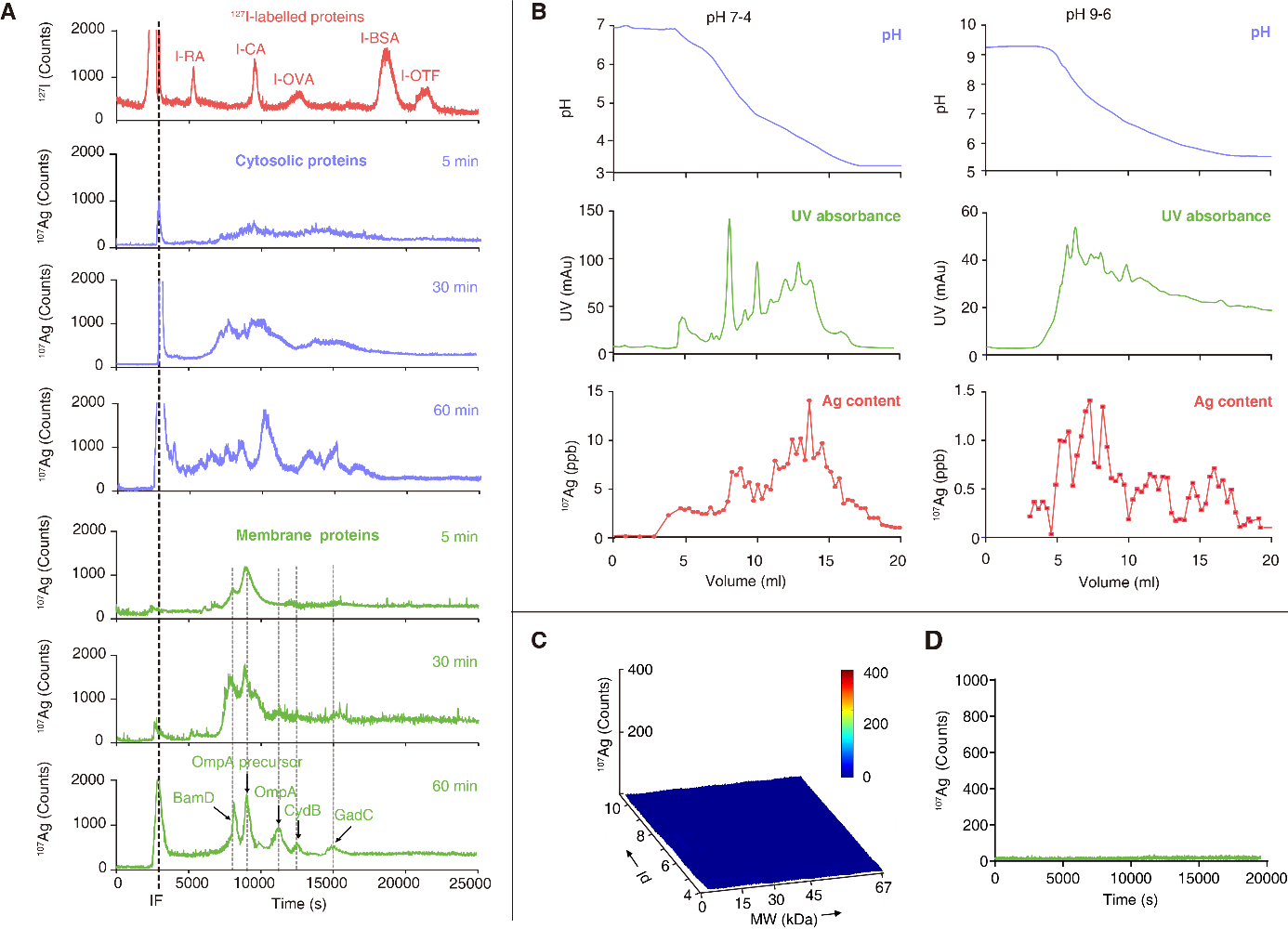

Supplement: S3 Fig — (A)1D GE-ICP-MS profiles of 127I-labeled standard proteins, Ag+-binding proteins in cytosol and membrane of E. coli at different time-points after treatment with 4 μg/ml AgNO3. (B) Separation of cytosolic proteins with LC (pH 7.4, left; pH 9.6, right). UV absorbance (280 nm), pH, and silver content are indicated. The silver contents and UV signals show a positive correlation. (C) Map of Ag+-associated proteins in the cytosol of E. coli without treatment with Ag+. (D) 1D GE-ICP-MS profile of Ag+-binding proteins in the membrane of E. coli without treatment with Ag+. No peaks corresponding to Ag+-binding proteins were observed in both cytosolic and membrane proteins. For all GE-ICP-MS experiments, one representative of three independent experiments is shown. Ag, silver; AgNO3, silver nitrate; GE, gel electrophoresis; ICP-MS, inductively coupled plasma mass spectrometry; LC, liquid chromatography. (TIF) [file pbio.3000292.s005.tif]

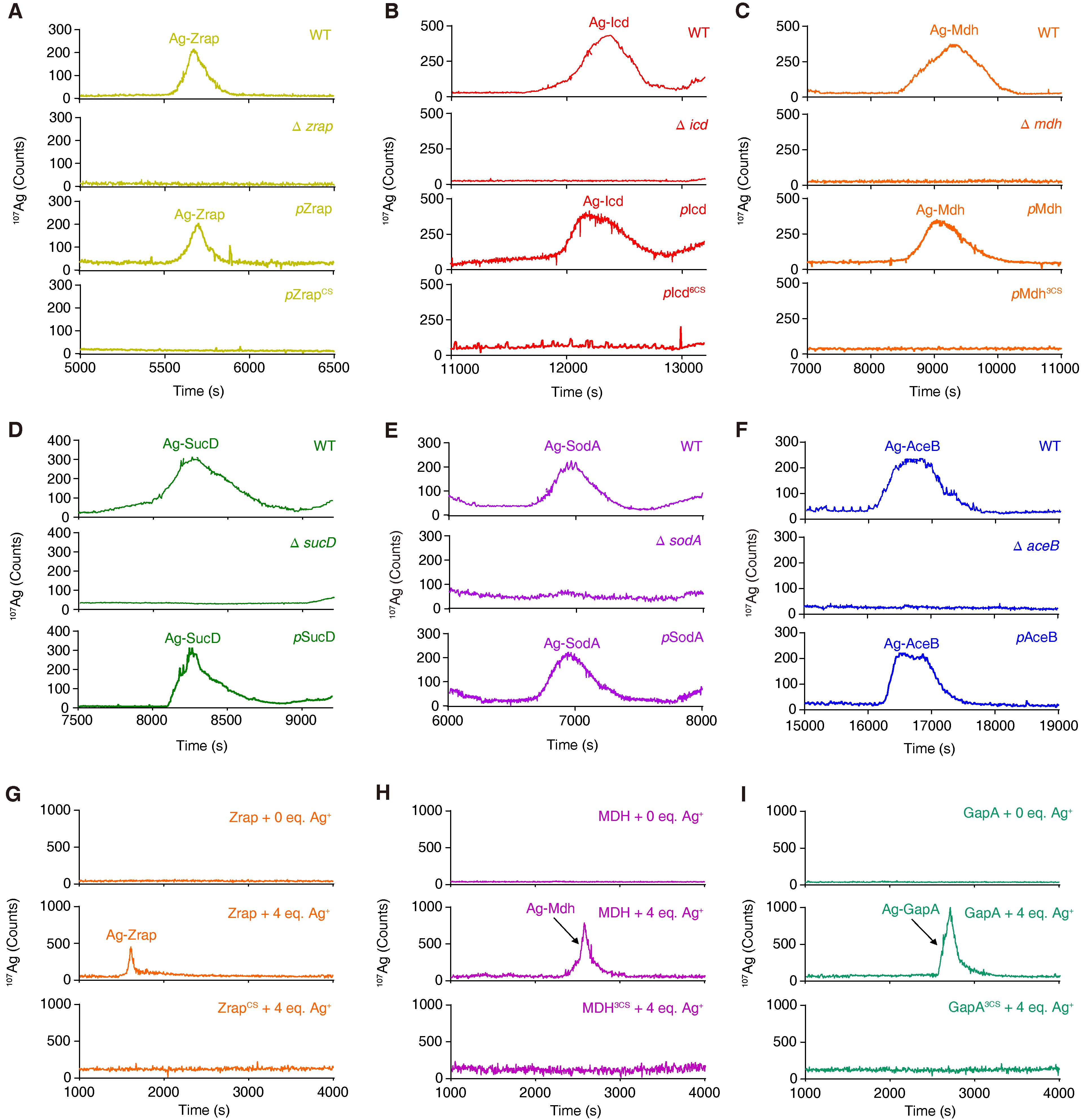

Supplement: S4 Fig — (A–F) Comparison of GE-ICP-MS profiles of Ag+-binding proteins in WT E. coli strain, its isogenic gene deletion mutants, and mutants containing the plasmids that express the corresponding genes (with or without site-specific mutagenesis). (A) Zrap. (B) Icd. (C) Mdh. (D) SucD. (E) SodA. (F) AceB. (G–I) GE-ICP-MS profiles of purified proteins with and without pre-incubation with Ag+. (G) Zrap. (H) Mdh. (I) GapA. Ag, silver; GE, gel electrophoresis; ICP-MS, inductively coupled plasma mass spectrometry; LC, liquid chromatography; WT, wild-type. (TIF) [file pbio.3000292.s006.tif]

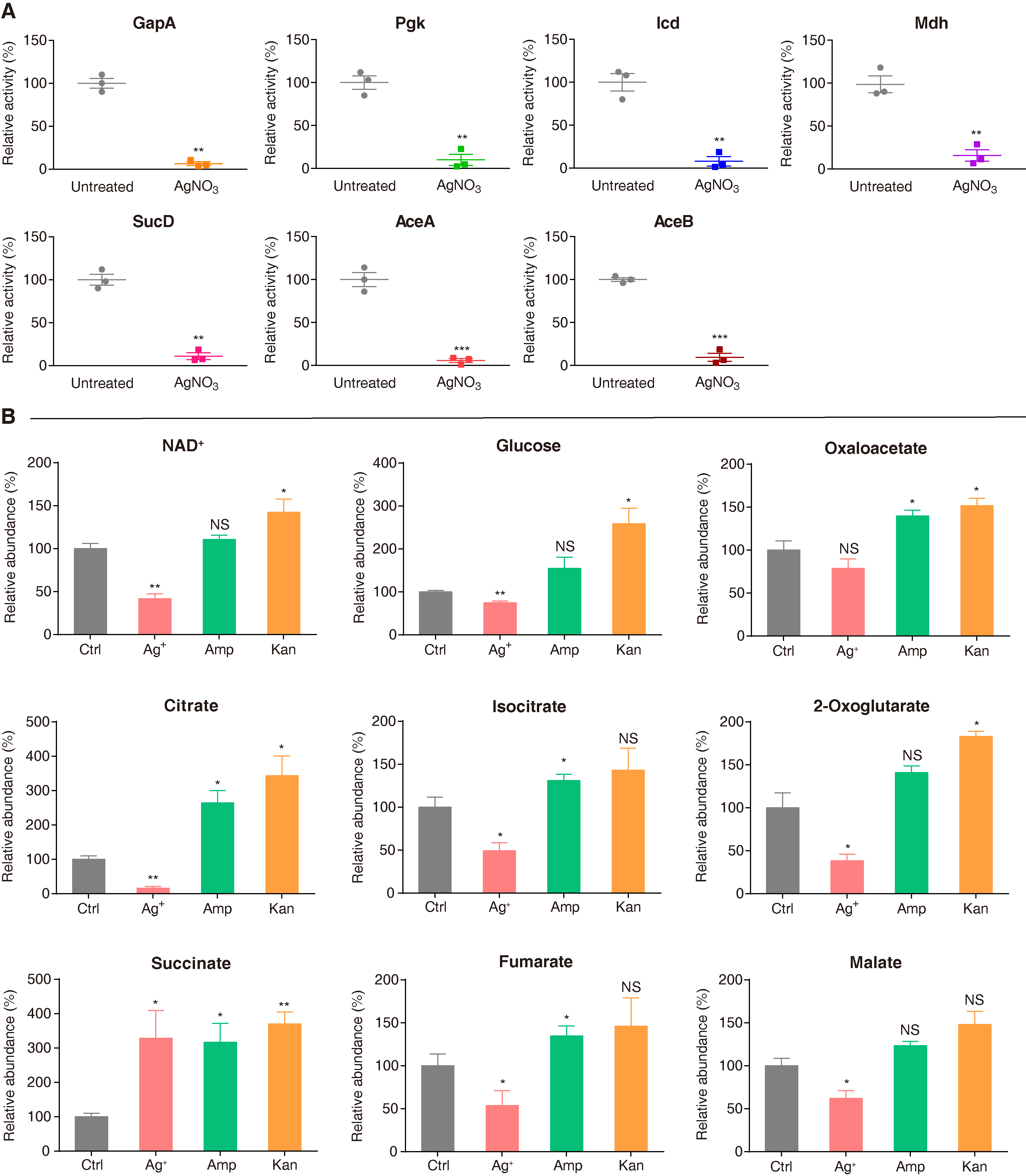

Supplement: S5 Fig — (A, B) Eight biological processes were significantly enriched by GO analysis (P < 0.05). Among them, TCA cycle is the one enriched with the highest significance (P = 5.2 × 10^−6^). Five identified Ag+-binding proteins are involved in TCA cycle. (C, D) Six signaling pathways were significantly over presented (P < 0.05). The TCA cycle is the one with the highest significance (P = 7.7 × 10^−5^). (E, F) Four cellular components were enriched. (G) Silver contained in identified proteins. (H) Distribution of silver in different pathways. (G, H) Representative results of three replicates. (I) PPIs. Ag, silver; GO, Gene Ontology; PPI, protein–protein interactions; TCA, tricarboxylic acid. (TIF) [file pbio.3000292.s007.tif]

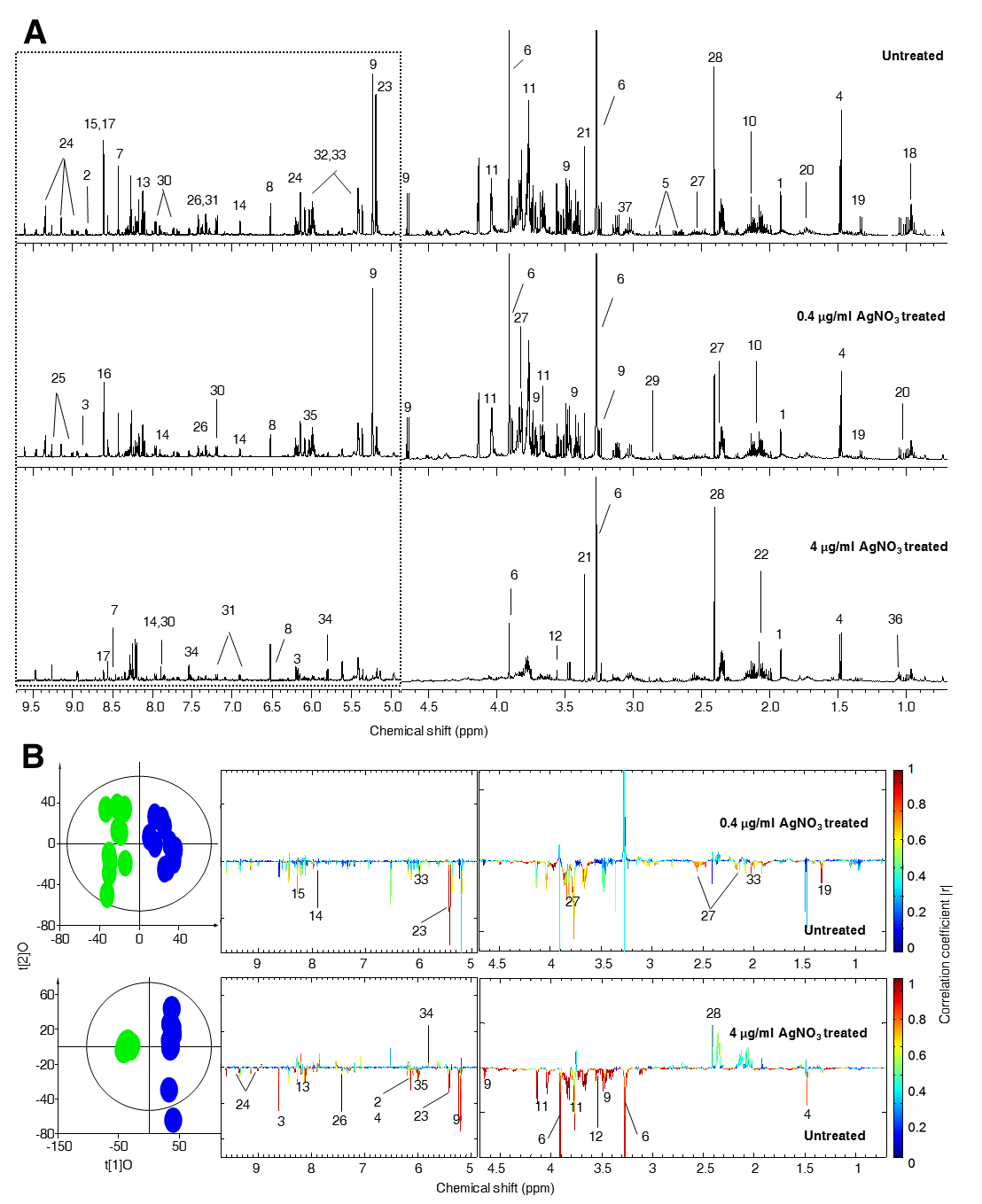

Supplement: S6 Fig — (A) Representative 600 MHz 1H NMR spectra of aqueous extracts from untreated, 0.4 μg/ml AgNO3-treated, and 4 μg/ml AgNO3-treated E. coli cells. Key: 1. Acetate; 2. Adenosine diphosphate (ADP); 3. Adenosine monophosphate (AMP); 4. Alanine (Ala); 5. Aspartate (Asp); 6. Betaine; 7. Formate; 8. Fumarate; 9. Glucose (Glc); 10. Glutamate (Glu); 11. Gluconate; 12. Glycine (Gly); 13. Guanosine; 14. Histidine (His); 15. Hypoxanthine; 16. Inosine; 17. Inosine-5'-monophosphate (5'-IMP); 18. Isoleucine (Ile); 19. Lactate; 20. Leucine (Leu); 21. Methanol; 22. Methionine (Met); 23. N-acetyl-glucosamine (GlcNAc); 24. Nicotinamide adenine dinucleotide (NAD+); 25. Nicotinamide adenine dinucleotide phosphate (NADP+); 26. Phenylalanine (Phe); 27. Reduced glutathione (GSH); 28. Succinate; 29. Trimethylamine (TMA); 30. Tryptophan (Trp); 31. Tyrosine (Tyr); 32. UDP glucuronate (UDP-GlcA); 33. UDP-N-acetyl glucosamine (UDP-GlcNAc); 34. Uracil; 35. Uridine; 36. Valine (Val); 37. Lysine (Lys). (B) O-PLS-DA scores plots (left) and coefficient-coded loading plots (right), discriminating between the untreated (green dots) and 0.4 μg/ml AgNO3 (low concentration) treated or 4 μg/ml AgNO3 (high concentration) treated E. coli cells (blue dots) (n = 10). These models are cross-validated with CV-ANOVA, P < 0.05. Metabolite keys corresponding to the numbers are shown in S6 Table. Detailed results of alterations in metabolites are presented in S7 Table. (TIF) [file pbio.3000292.s008.tif]

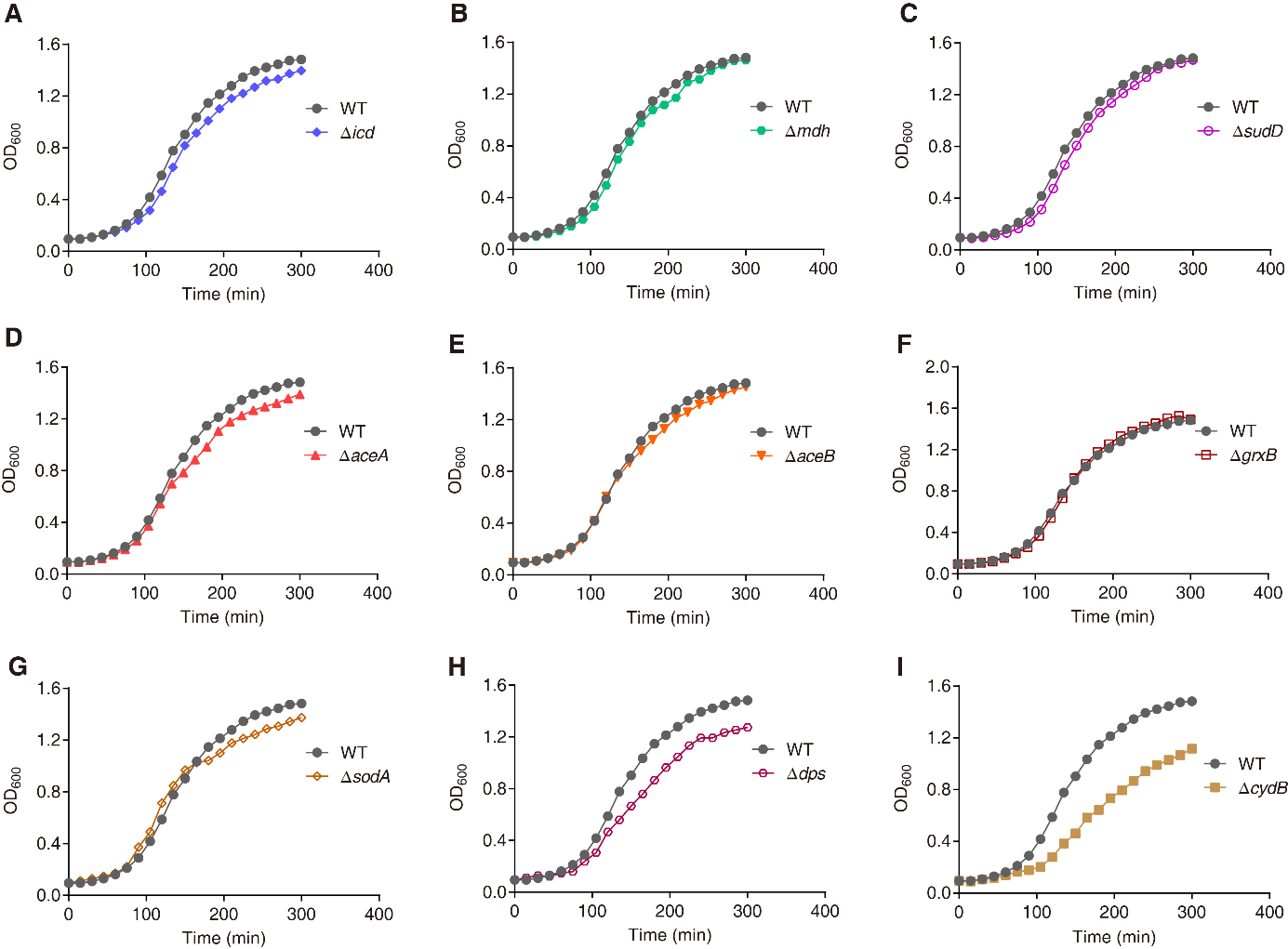

Supplement: S8 Fig — (A) Δicd, (B) Δmdh, (C) ΔsucD, (D) ΔaceA, (E) ΔaceB, (F) ΔgrxB, (G) ΔsodA, (H) Δdps, (I) ΔcydB. Numerical values that underlie the graphs are shown in S1 Data. WT, wild-type. (TIF) [file pbio.3000292.s010.tif]

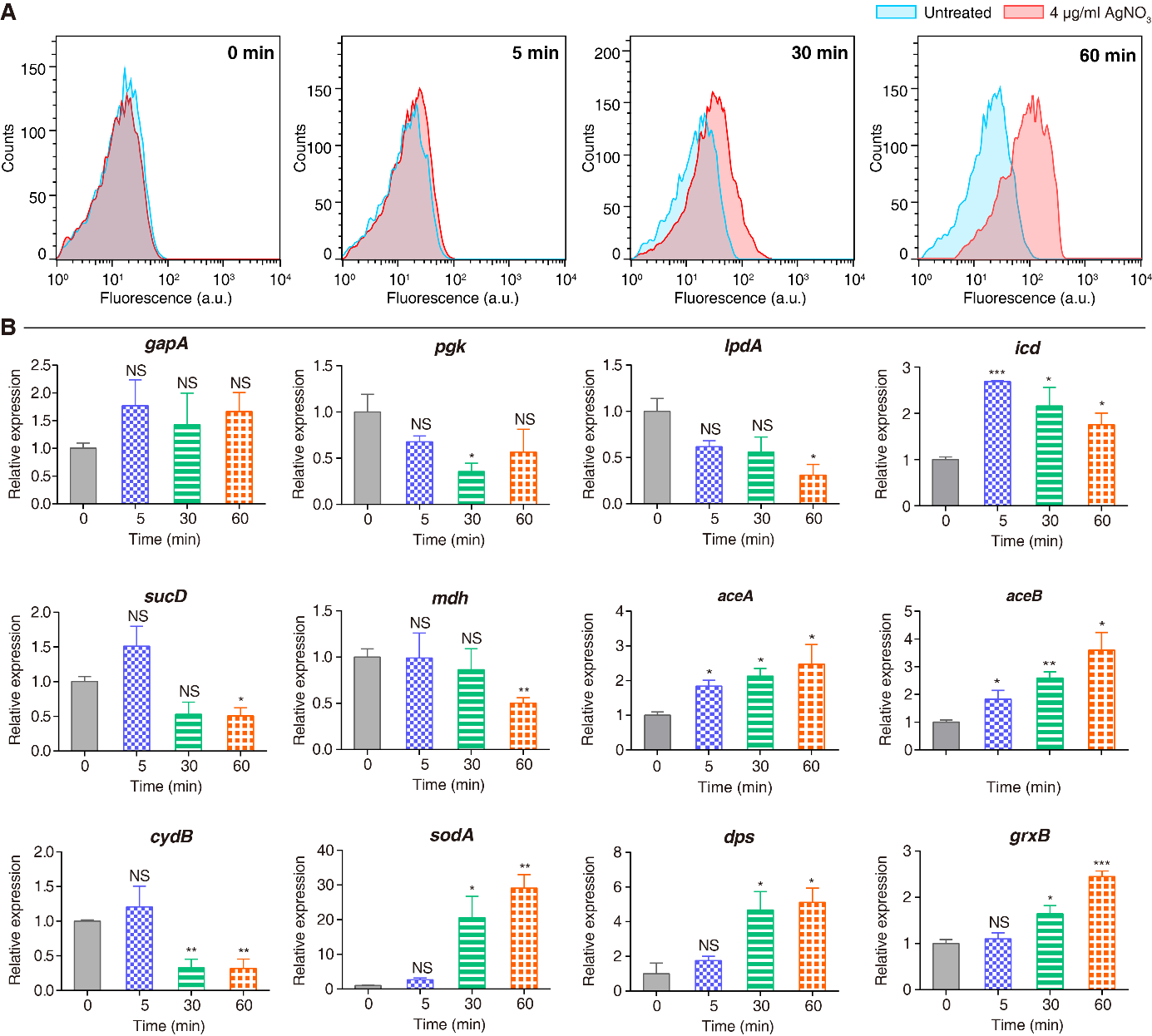

Supplement: S9 Fig — (A) CM-H2DCFDA fluorescence histogram of E. coli with (red) or without (blue) treatment of Ag+ (n = 3). Data are representatives of three replicates. (B) Relative gene expressions in E. coli treated with 4 μg/ml AgNO3 at different time points (n = 3). Gene expression was determined by qPCR and normalized against rrsA and untreated control. Two-tailed t test was used for all comparisons between two groups. Data are presented as mean ± SEM. *P < 0.05, ** P < 0.01, and *** P < 0.001. NS (P > 0.05). Numerical values that underlie the graphs are shown in S1 Data. Ag, silver; CM-H2DCFDA, chloromethyl derivative of 2′, 7′-dichlorodihydrofluorescein diacetate; NS, not significant; qRT-PCR, real-time quantitative polymerase chain reaction. (TIF) [file pbio.3000292.s011.tif]

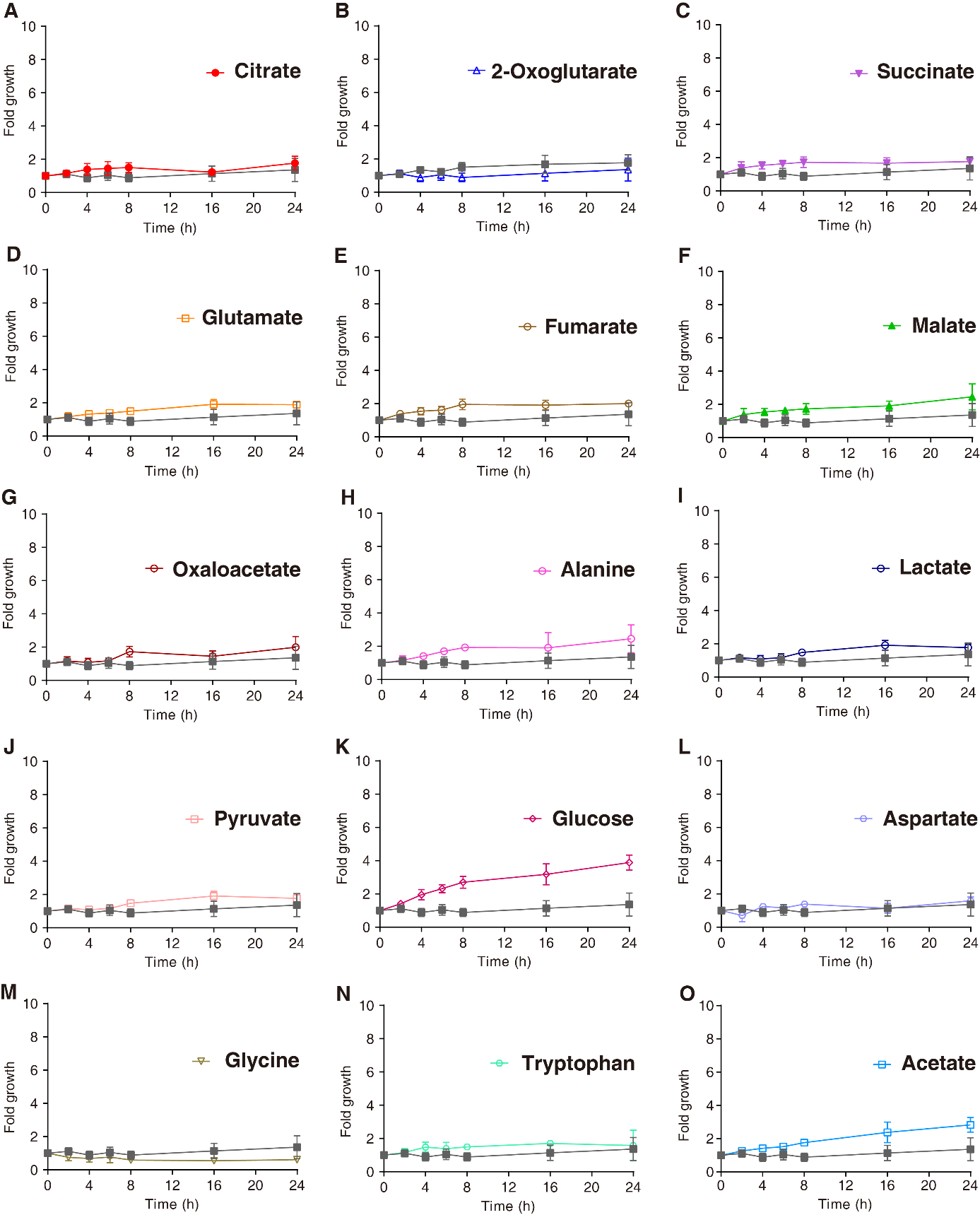

Supplement: S10 Fig — Gray lines represent untreated E. coli. (A) 50 mM citrate. (B) 50 mM 2-oxoglutarate. (C) 50 mM succinate. (D) 50 mM glutamate. (E) 50 mM fumarate. (F) 50 mM malate. (G) 50 mM oxaloacetate. (H) 50 mM alanine. (I) 50 mM lactate. (J) 50 mM pyruvate. (K) 50 mM glucose. (L) 10 mM aspartate. (M) 50 mM glycine. (N) 10 mM tryptophan. (O) 50 mM acetate. For each experiment, three biological replicates were performed. Data are presented as mean ± SEM. Numerical values that underlie the graphs are shown in S1 Data. (TIF) [file pbio.3000292.s012.tif]

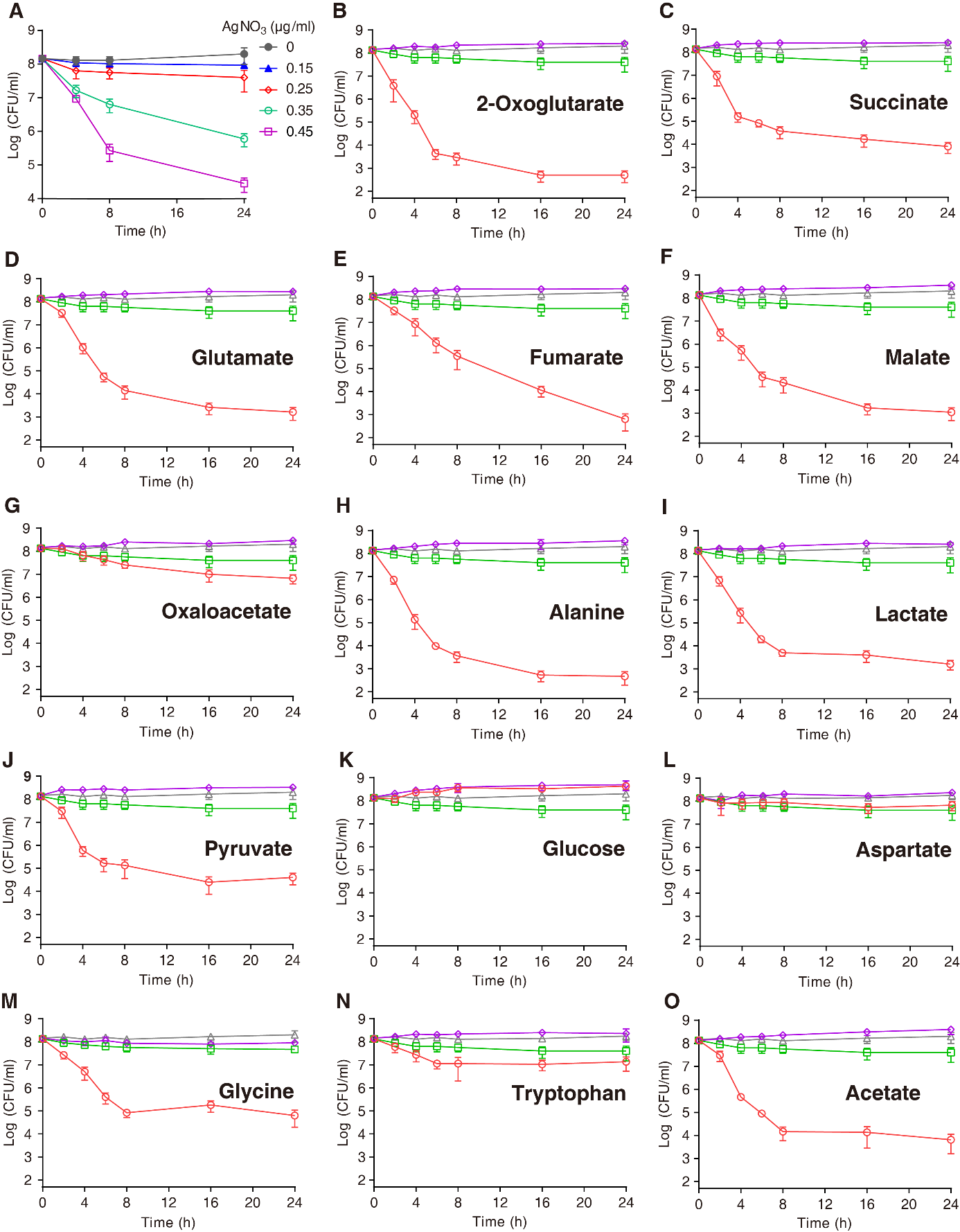

Supplement: S11 Fig — Gray, purple, green, and red lines represent control group; metabolite treated; 0.25 μg/ml AgNO3 treated; 0.25 μg/ml AgNO3 together with metabolite treated E. coli cells, respectively. (A) Survival of E. coli after treatment of different concentration of AgNO3 in M9 minimal medium. (B) 50 mM 2-oxoglutarate. (C) 50 mM succinate. (D) 50 mM glutamate. (E) 50 mM fumarate. (F) 50 mM malate. (G) 50 mM oxaloacetate. (H) 50 mM alanine. (I) 50 mM lactate. (J) 50 mM pyruvate. (K) 50 mM glucose. (L) 10 mM aspartate. (M) 50 mM glycine. (N) 10 mM tryptophan. (O) 50 mM acetate. For each experiment, three biological replicates were performed. Data are presented as mean ± SEM. Numerical values that underlie the graphs are shown in S1 Data. Ag, silver; AgNO3, silver nitrate. (TIF) [file pbio.3000292.s013.tif]

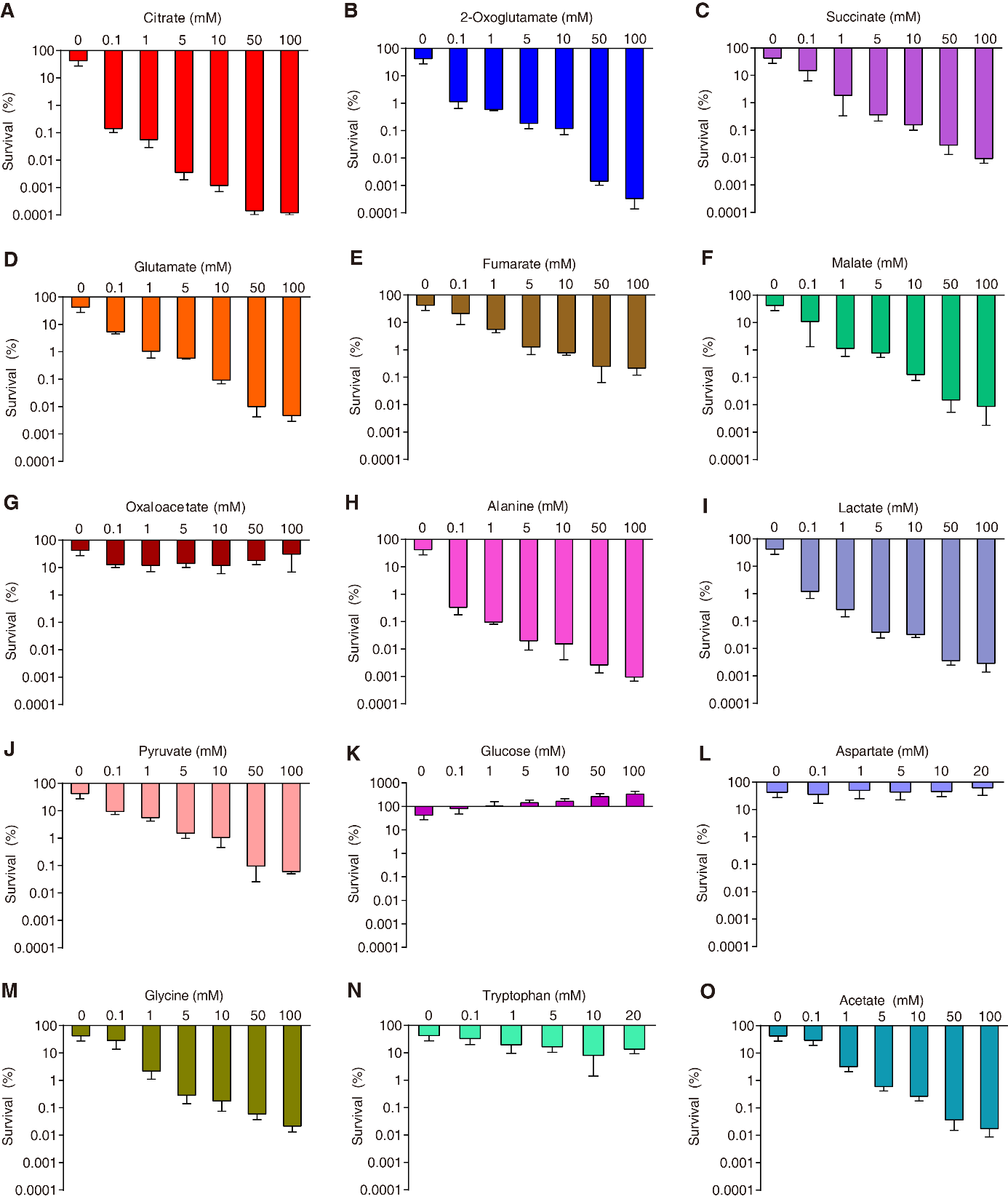

Supplement: S12 Fig — E. coli cells were coadministered with 0.25 μg/ml AgNO3 and different metabolites for 8 hrs. (A) Citrate. (B) 2-Oxoglutarate. (C) Succinate. (D) Glutamate. (E) Fumarate. (F) Malate. (G) Oxaloacetate. (H) Alanine. (I) Lactate. (J) Pyruvate. (K) Glucose. (L) Aspartate. (M) Glycine. (N) Tryptophan. (O) Acetate. For each experiment, three biological replicates were performed. Data are presented as mean ± SEM. Numerical values that underlie the graphs are shown in S1 Data. Ag, silver; AgNO3, silver nitrate. (TIF) [file pbio.3000292.s014.tif]

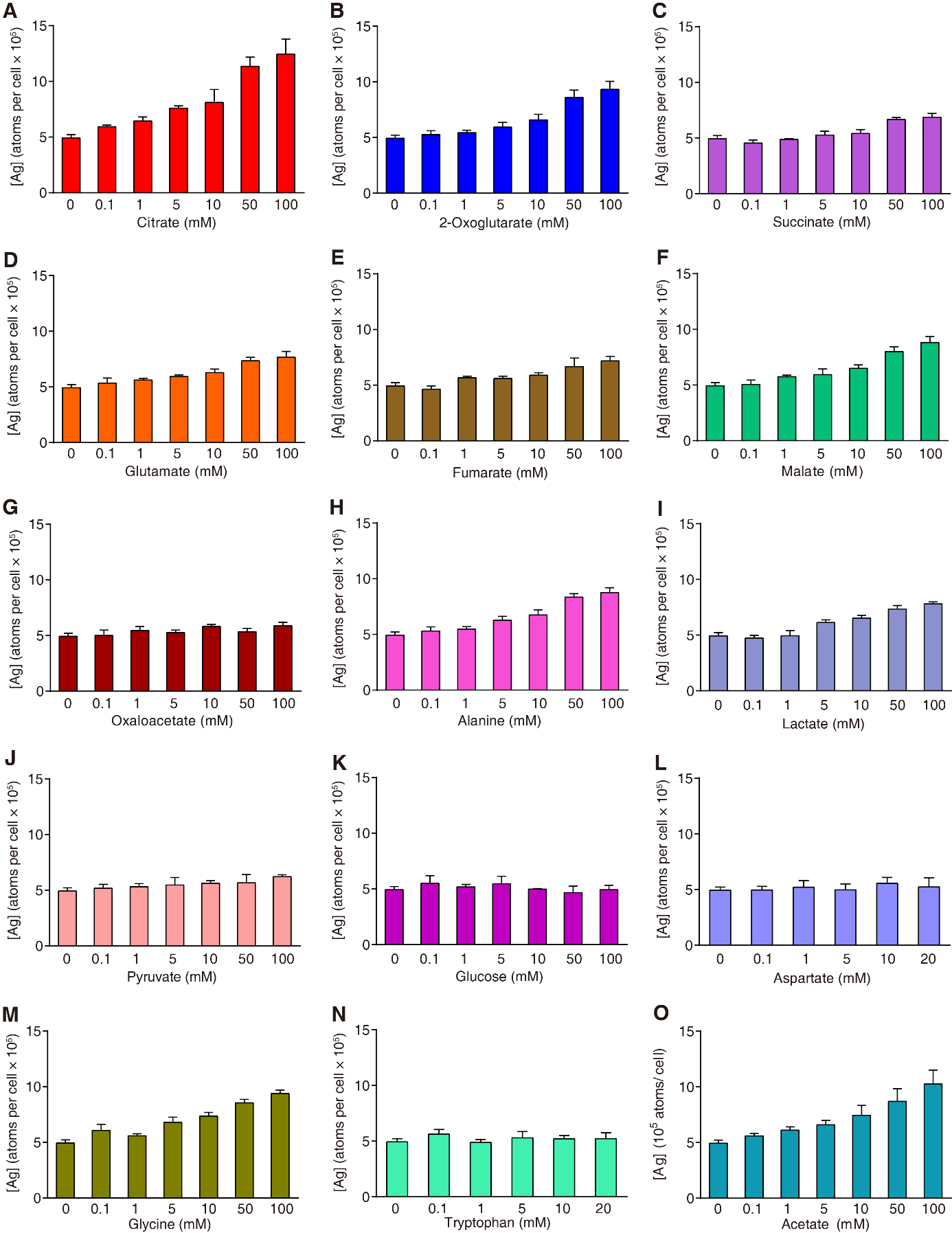

Supplement: S13 Fig — E. coli cells were coadministered with 0.25 μg/ml AgNO3 and different metabolites for 1 h. (A) Citrate. (B) 2-Oxoglutarate. (C) Succinate. (D) Glutamate. (E) Fumarate. (F) Malate. (G) Oxaloacetate. (H) Alanine. (I) Lactate. (J) Pyruvate. (K) Glucose. (L) Aspartate. (M) Glycine. (N) Tryptophan. (O) Acetate. For each experiment, three biological replicates were performed. Data are presented as mean ± SEM. Numerical values that underlie the graphs are shown in S1 Data. Ag, silver; AgNO3, silver nitrate. (TIF) [file pbio.3000292.s015.tif]

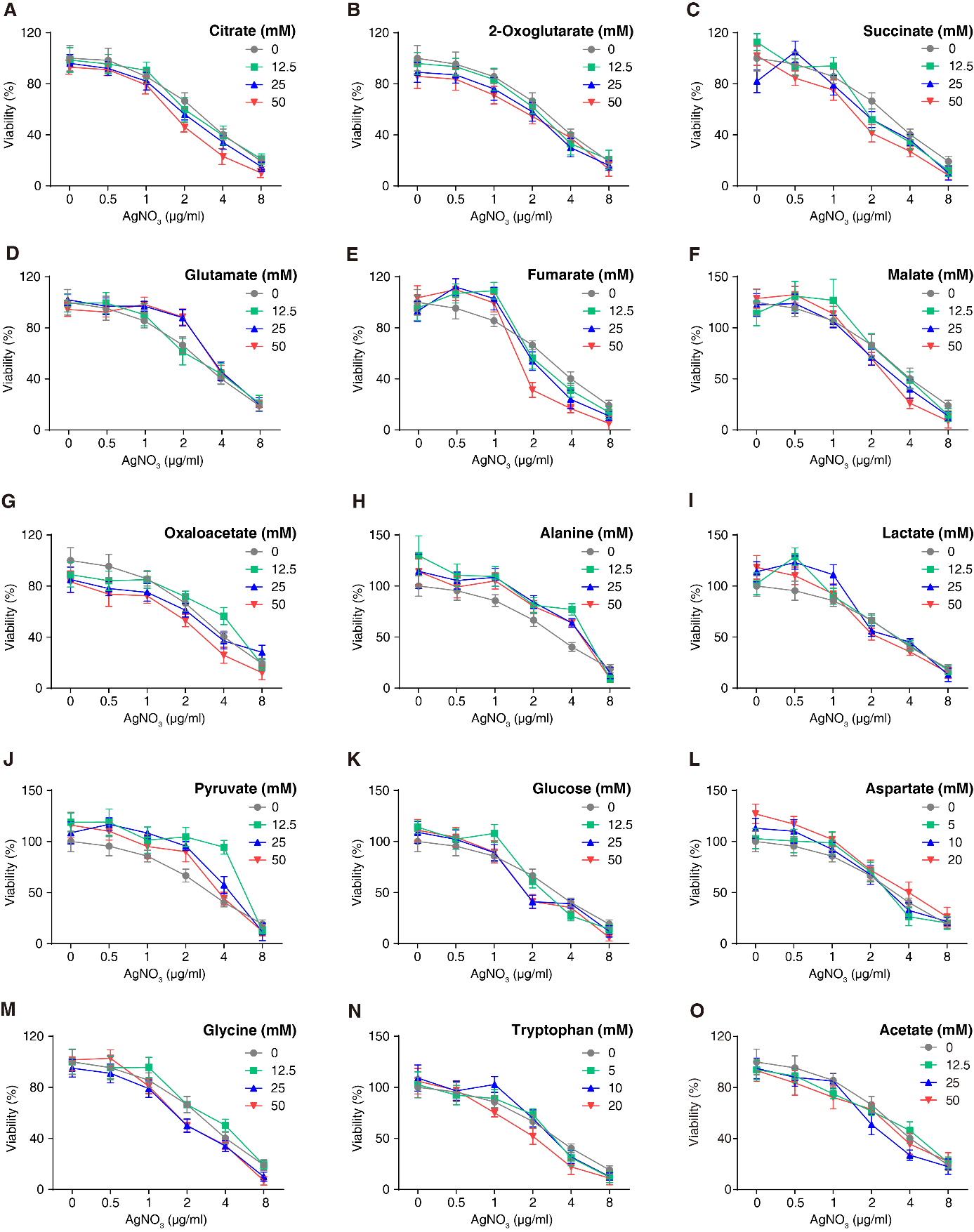

Supplement: S14 Fig — HeLa cells were treated with serial concentrations of Ag+ and metabolites for 24 h. The cell toxicity was detected by Cell Proliferation Kit (XTT). All the metabolites showed no enhancement on the toxicity of Ag+ to human HeLa cells. (A) Citrate. (B) 2-Oxoglutarate. (C) Succinate. (D) Glutamate. (E) Fumarate. (F) Malate. (G) Oxaloacetate. (H) Alanine. (I) Lactate. (J) Pyruvate. (K) Glucose. (L) Aspartate. (M) Glycine. (N) Tryptophan. (O) Acetate. For each experiment, three biological replicates were performed. Data are presented as mean ± SEM. Numerical values that underlie the graphs are shown in S1 Data. Ag, silver; HeLa, human epithelial. (TIF) [file pbio.3000292.s016.tif]

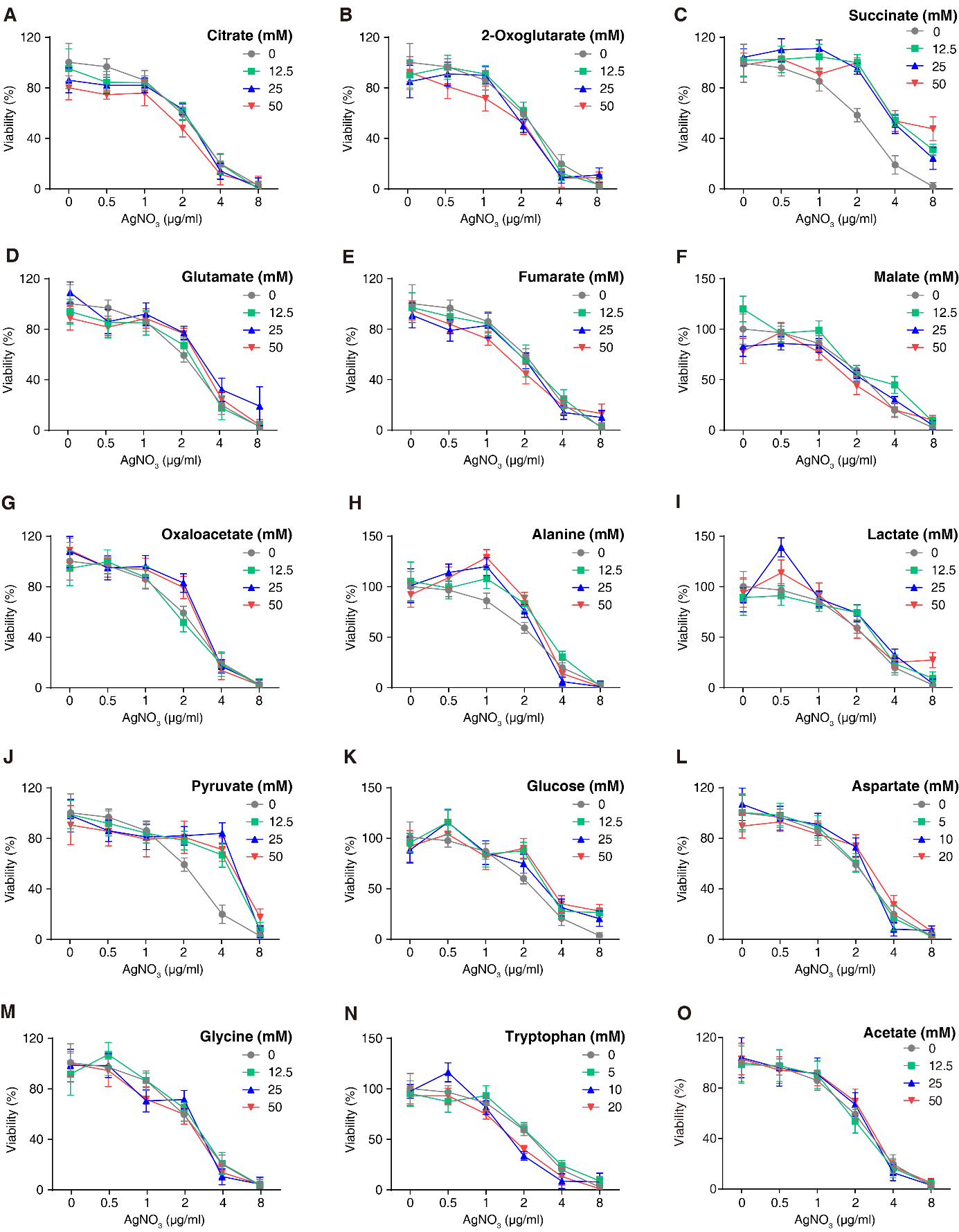

Supplement: S15 Fig — HepG2 cells were treated with serial concentrations of Ag+ and metabolites for 24 h. The cell toxicity was detected by Cell Proliferation Kit (XTT). All the metabolites showed no enhancement on the toxicity of Ag+ to human HeLa cells. (A) Citrate. (B) 2-Oxoglutarate. (C) Succinate. (D) Glutamate. (E) Fumarate. (F) Malate. (G) Oxaloacetate. (H) Alanine. (I) Lactate. (J) Pyruvate. (K) Glucose. (L) Aspartate. (M) Glycine. (N) Tryptophan. (O) Acetate. For each experiment, three biological replicates were performed. Data are presented as mean ± SEM. Numerical values that underlie the graphs are shown in S1 Data. Ag, silver; HeLa; human epithelial; HepG2, human hepatoma G2. (TIF) [file pbio.3000292.s017.tif]
